# Challenging the Public Stigma of Alcohol Use Disorder in the UK Using Video ‘Education’ and ‘Contact’ Interventions: A Pilot Study

**DOI:** 10.1007/s11469-024-01393-y

**Published:** 2024-10-09

**Authors:** Sophie Hytner, Daphne Josselin, David Belin, Owen Bowden Jones

**Affiliations:** 1 Department of Psychology, University of Cambridge, Cambridge, CB2 3EB UK; 2 Department of Psychology, City, University of London, Northampton Square, London, EC1V 0HB UK; 3 Department of Clinical, Educational and Health Psychology, University College London, 1-19 Torrington Pl, London, WC1E 7HB UK

**Keywords:** Public Stigma, UK, Alcohol Use Disorder, Anti-Stigma Interventions, Pilot, Feasibility

## Abstract

Public stigma negatively impacts people with alcohol use disorder; yet, few interventions exist to reduce it. This quasi-randomised controlled pilot study aimed to evaluate the feasibility and efficacy of three such interventions. Participants (539 UK-based adults) watched a video presenting myths and facts about AUD (‘education’), personal testimonies about AUD (‘contact’), a combination of both (‘education and contact’) or a control video. Feasibility was assessed by examining recruitment capability, study retention and data quality, and participant satisfaction with the study’s procedures and interventions. Efficacy was assessed using self-report, public stigma measures issued at pre-test, post-test and 1-month follow-up. Stigma reduced significantly more in the intervention groups than in the control group at post-test and follow-up. Feasibility was generally high, though could be improved by shortening video length and enhancing video production quality. Education and contact videos are promising interventions for public AUD stigma, and merit further investigation.

**Supplementary Information:**

The online version contains supplementary material available at 10.1007/s11469-024-01393-y.

Stigma is defined as a socially discrediting attribute, making an individual ‘different’ and ‘less desirable’ to others (Goffman, 1963). Public stigma of alcohol use disorder (AUD) is prevalent and resistant to change in the UK and other countries (Crisp et al., 2005; Kilian et al., 2021; Schomerus et al., 2014). Among people with AUD, this negatively impacts psychological wellbeing (Glass et al., 2013; Hunter et al., 2017; Smith et al., 2010), treatment seeking (Finn et al., 2023; Keyes et al., 2010; Probst et al., 2015; Wallhed Finn et al., 2014) and recovery (Crapanzano et al., 2018; Luoma et al., 2014; Mak et al., 2017).

There is a need for more trials on public anti-stigma interventions for AUD. Public stigma underpins other forms of stigma (Corrigan et al., 2011; Evans-Lacko et al., 2012; Pryor & Reeder, 2011; Schomerus et al., 2011) and can potentially be countered with briefer, more scalable interventions. Yet, existing anti-stigma intervention research for substance use disorders focuses on self- and structural stigma, with only three studies hitherto targeting public stigma (Livingston et al., 2012; Tostes et al., 2020). These provided limited intervention descriptions and investigated short-term outcomes without control groups (Luty et al., 2007, 2008, 2009), preventing attribution of observed changes to the interventions implemented. The present study seeks to address this gap by evaluating anti-stigma interventions for AUD. Given the limited research available on addiction-specific anti-stigma interventions, the interventions were developed based on the more extensively researched area of mental illness stigma (Corrigan et al., 2017), in line with the Medical Research Council’s framework for developing and evaluating interventions (Skivington et al., 2021). These typically rely on three strategies: protest, education and contact.

Protest highlights injustices of stigma, contact facilitates interactions between ingroups (e.g., the public) and outgroups (e.g., people with neuropsychiatric disorders), and education provides information that challenges stigmatising beliefs (Corrigan, 2015). While protest can have unintended rebound effects, contact and education demonstrate individual and combined effectiveness (Corrigan et al., 2012; Griffiths et al., 2014; Morgan et al., 2018; Thornicroft et al., 2016), though their relative effectiveness is unclear. Contact was previously recognised as superior to education (Corrigan et al., 2012), but recent evidence suggests no clear advantage of either and similar effects between combined and sole interventions (Morgan et al., 2018).

Accordingly, this pilot study gathers preliminary evidence for the immediate and sustained relative efficacy of different interventions, namely education, contact and combined education and contact, in reducing the UK public stigma of AUD. To lay the foundations for a larger future trial, this study aimed to assess the feasibility of both the procedures implemented and the nature of each intervention (Skivington et al., 2021).

Brief videos were used as these allow high intervention fidelity and easy dissemination at reduced cost while offering comparable effectiveness to face-to-face mediums (Maunder & White, 2019; Morgan et al., 2018). Aiming to tackle stigma of severe AUD, the study used the term alcohol dependence with participants (Saunders et al., 2019) to delineate severe alcohol problems with accessible language.

The interventions’ expected effects were underpinned by Link and Phelan’s (2001) theory of stigma, which holds that stigmatisation takes place when an individual’s human differences are labelled (e.g., ‘alcoholic’) and, through dominant cultural beliefs, linked to undesirable stereotypes (e.g., ‘lazy’). These in turn mark the individual as different and lead them to experience status loss and discrimination. By linking people with AUD to counter-stereotypic attributes, the videos were expected to disrupt this stigmatisation process and reduce stigma.

The education video used a commonly used myth-fact format (Corrigan & Shapiro, 2010), which drew on attribution theory to influence participants feelings and behaviours by challenging inaccurate stereotypes about AUD with facts (Weiner, 1986). Drawing on contact theory, the contact video sought to disconfirm stereotypes of AUD by using indirect contact with people with past experience of AUD sharing their stories (Allport, 1954). ‘Moderate disconfirmation’ was favoured over ‘strong disconfirmation’ to align with the model of stereotype change (Rothbart & John, 1985), which holds that strongly disconfirming stories (e.g., a sole focus on recovery) lead ingroup members to subtype the counterstereotypic group member as ‘deviant’, blocking generalisation to the outgroup as a whole. Meanwhile, moderately disconfirming stories (e.g., that also describe symptoms and challenges) have been shown to facilitate greater stereotype change (Reinke et al., 2004).

Based on existing literature and theoretical frameworks, the study’s hypotheses were as follows:

- H1: There will be a significant difference in mean stigma scores between groups, which will depend on the interaction between time-point and intervention group
- H2: There will be a significant difference in the intervention groups’ mean stigma scores at the different time-points (pre-test, post-test and follow-up) vs. the control group

## Methods

### Design

This study was a quasi-randomised controlled pilot trial of parallel groups (three anti-stigma interventions and a control) with repeated stigma measurement at pre-test, post-test and follow-up. Participants were recruited and paid through the online crowd-sourcing platform Prolific. Inclusion criteria were 18 + , English-speaking, living in the UK and consenting to take part. Individuals with experience of AUD (both personal and indirect) were able to participate. Quasi-random allocation into groups was achieved by running four sequential recruitment phases (one for each group), each closing before the next opened and showing identical recruitment information to ensure participants were blinded. Each group was capped at 150 completing participants, with repeat enrolment barred via Prolific.

### Procedures

City, University of London’s Ethics Committee approved the study, which comprised two stages. In stage one, eligible participants who provided informed consent completed demographic information and stigma measures before individually watching either an education (EV), contact (CV), combined education and contact (CombV) or control (CtrlV) video online. They then answered an attention check question, repeated the measures and completed feasibility questions before viewing debrief information with support resources. At a 1 month follow-up (stage two), participants were invited via Prolific IDs to repeat the measures. Participants from all rounds then received further debrief information. Materials and interventions were hosted via Prolific on the online survey platform Qualtrics and on the video-hosting platform MediaSpace.

### Measures

#### Demographic

Participants completed demographic information, including their age, gender, ethnicity and experience (direct and indirect) of AUD.

#### Outcome Measures

**Fig. 1:**
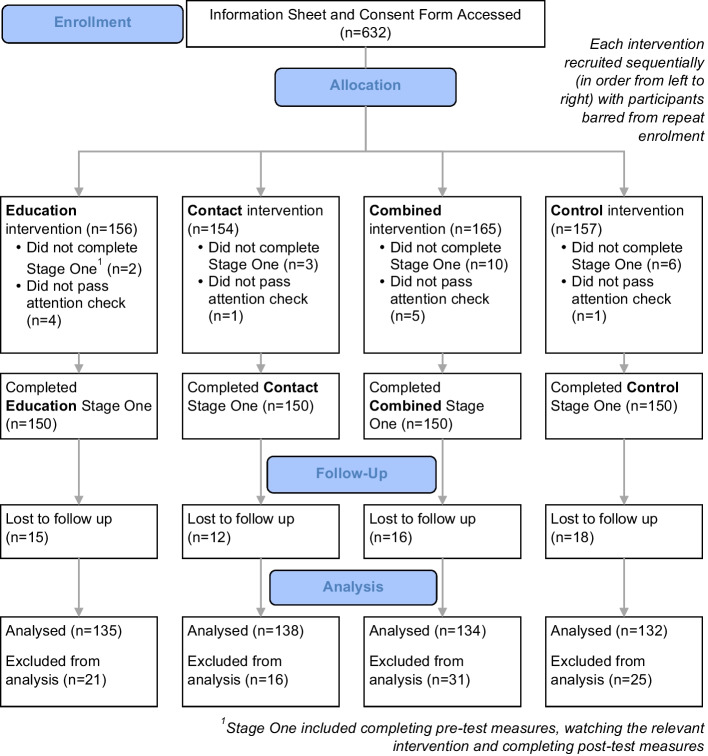
Consort flow diagram

Two self-report measures of public mental illness stigma were used: the Attribution-Questionnaire-27 (AQ-27) and the social distance scale (SDS). Both include items about a vignette subject with mental illness. Our study used a modified vignette with both, like other studies examining addiction-related stigma (Abdullah & Brown, 2020; Abraham et al., 2013; Janulis et al., 2013; Pescosolido et al., 2010). This depicted a man (‘John’) with symptoms meeting DSM-IV criteria for alcohol dependence (American Psychiatric Association, 2000), and is presented in Supplementary Online Materials (SOM) Figure 1.

AQ-27*.* The AQ-27 includes 27 items representing nine subscales: blame, anger, pity, help, dangerousness, fear, avoidance, segregation and coercion. Participants rate items on a 9-point Likert scale ranging from 1 (low agreement) to 9 (high agreement) (Corrigan, 2008). Scores are summed to create an AQ score (27 to 243). Higher scores indicate higher stigma. The AQ-27 demonstrates reliability and validity (Pinto et al., 2012), demonstrating good test–retest reliability across subscales (*r* > 0.75) and convergent validity with social distance measures (Brown, 2008).

SDS*.* The SDS measures intentions to distance socially from the vignette subject (Link, 1987; Penn et al., 1994). Its seven items are rated on a 4-point Likert scale ranging from 0 (definitely willing) to 3 (definitely unwilling). Responses are summed and divided by seven to generate a SDS score (0 to 3). Higher scores indicate greater stigma. The SDS demonstrates validity and good reliability, with internal consistency ranging from *α* = 0.75 to *α* = 0.92 (Link, 1987; Wei et al., 2015).

Feasibility Questionnaire*.* A feasibility questionnaire was developed (SOM Table I). This contained eleven quantitative and two qualitative questions assessing Orsmond and Cohn’s (2015) feasibility research objectives, such as evaluating the suitability of study procedures. Participants ranked most items using a 5-point Likert scale ranging from strongly disagree (1) to strongly agree (5), with items about procedure duration ranked on a 3-point Likert scale ranging from too short (1) to too long (3).

### Interventions

#### Education

Following a typical myth-fact structure (Corrigan et al., 2001), the 4-min education video countered five common myths about AUD: that AUD only affects certain groups, and that people with AUD cannot recover, are to blame for their problems, can control their drinking and don’t care about others.

Myth-fact pairs were developed from a Delphi study where UK-based experts in AUD (five academics, five clinicians and eight experts-by-experience) listed common myths and facts about AUD and rated their importance for inclusion in an anti-stigma intervention. The video was an animated explainer created with an animation software (Vyond), since this medium benefits learning and engagement (Berney & Bétrancourt, 2016; Höffler & Leutner, 2007; Shahbaznezhad et al., 2021).

Using theory to guide intervention development optimises effectiveness (Craig et al., 2008; Michie et al., 2011). Attribution theory guided the education video, which holds that attributions about a person with a stigmatised condition (e.g., ‘he is to blame for his AUD’) shape affective (e.g., anger) and behavioural responses (e.g., diminished helping behaviour) (Jones, 1984; Weiner, 1986). It was expected that by challenging stigmatising attributions, the video would improve participants’ feelings and intended behaviour towards people with AUD.

A copy of the video is available on request. SOM Table II presents its content.

#### Contact

The 4-min contact video showed clips from online recorded interviews with three public speakers sharing personal experiences of AUD. Speakers were sent recruitment information via social media before consenting to interviews. They comprised two males and one female, aged 40–60 years old, with white (× 2) and mixed white and black Caribbean (× 1) ethnicities.

The interview guide was based on a systematic review of common themes in effective contact interventions for public stigma of neuropsychiatric disorders. It comprised: Section 1: introductions, background, symptoms, challenges; Section 2: acceptance/treatment, recovery, achievements; and Section 3: ongoing challenges and hope.

The contact hypothesis guided the video. This proposes that contact between ingroups (e.g., the public) and outgroups (e.g., people with AUD) reduces ingroup prejudice through enhanced knowledge (Allport et al., 1979). Balancing the discussion of symptoms and challenges with successes indicative of recovery was intended to moderately disconfirm AUD-related stereotypes (Rothbart & John, 1985). While certain other ‘optimal’ conditions, like group cooperation, may enhance contact effects, these are not necessary for effectiveness (Pettigrew & Tropp, 2006). It was therefore expected that mere exposure to the outgroup would reduce stigma.

SOM Table III presents the interview guide and video content.

#### Combined

The 8-min combined video presented the education and contact interventions in sequence, with education first to enable deeper processing of combined content (Chan et al., 2009).

#### Control

The 4-min control video presented educational content about tornadoes.

### Analyses

Data were analysed using SPSS. Statistical significance for tests was defined at a 95% confidence level and *α* = 0.05.

A priori power analysis was conducted using G power. No studies with comparable research design were retrieved. A significance criterion of *α* = 0.05 and power = 0.80 meant the minimum sample needed was 100 with a medium effect size and 460 with a small effect size (Cohen, (1988). Accordingly, 600 participants were targeted for stage one, providing contingency against a smaller effect size and dropout before follow-up, typically 20–40% in comparable studies (Keith et al., 2017).

Participants failing attention checks were excluded from analysis.

Boxplots were inspected for outliers greater than 1.5 × the interquartile range (or greater than 3 × the interquartile range for extreme outliers) above the third quartile or below the first quartile. Shapiro–Wilk, Levene’s and Mauchly’s tests were run to check assumptions of normality, homogeneity of variance and sphericity required for parametric analyses.

**Fig. 2:**
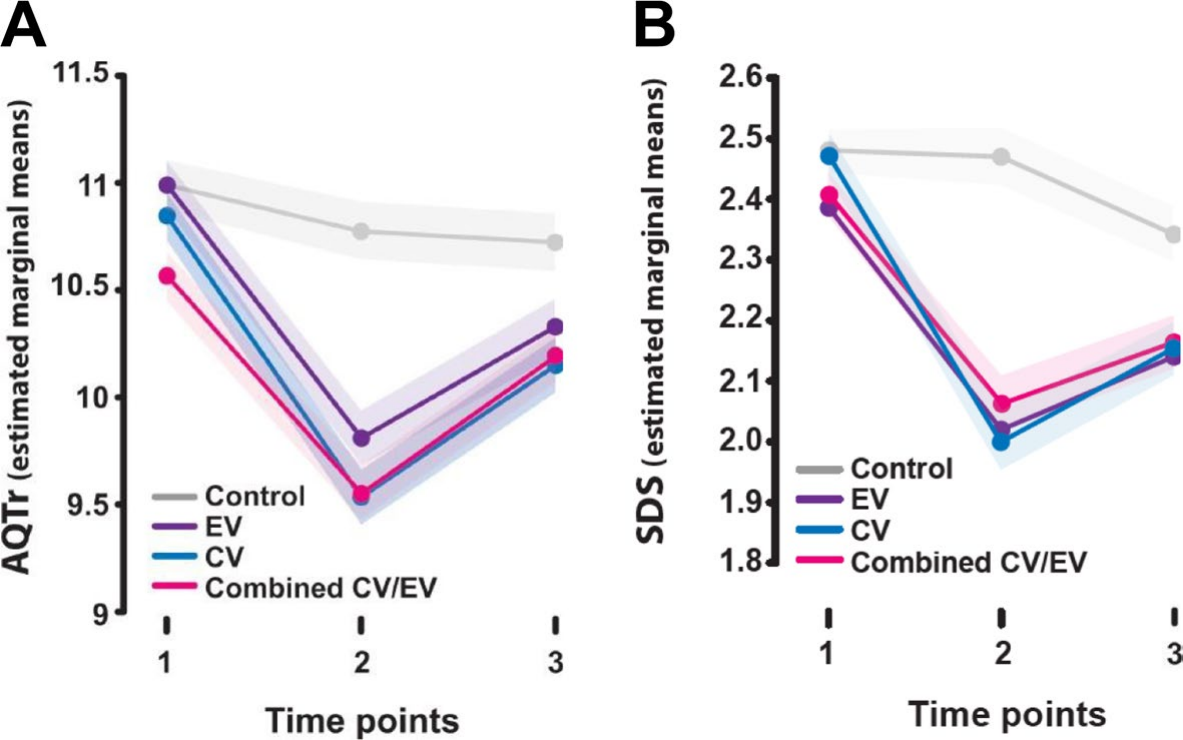
All interventions were effective in reducing participant stigma relative to a control group. **A**, **B** Participants watching an intervention video showed significantly reduced stigma relative to the control group immediately after watching the video at post-test (time point 2, all *p*s < 0.01). The impact reduced at follow-up (time point 3), but remained significantly lower than pre-test (timepoint 1) and relative to the control group for all interventions (all *p*s < 0.05). All intervention types were found to have broadly comparable impact, with minimal difference in change in stigma scores at either post-test or follow-up (all *p*s > 0.31)

Thirty-three outliers were identified (12 SDS below the threshold; 21 AQ above the threshold) (SOM Figure 2). One extremely low outlier in the SDS was included in the analysis since statistical tests did not yield significantly different results when it was included versus excluded.

Tests of normality are in SOM Figure 3. A Shapiro–Wilk test found the SDS distribution departed significantly from normality at pre-test, *W* (539) = 0.94, *p* ≤ 0.001; post-test, *W* (539) = 0.96, *p* ≤ 0.001; and follow-up, *W* (539) = 0.97, *p* ≤ 0.001, as did the distribution of the AQ at pre-test, *W* (539) = 0.99, *p* ≤ 0.005; post-test, *W* (539) = 0.98, *p* ≤ 0.001; and follow-up, *W* (539) = 0.99, *p* ≤ 0.001. This was corroborated by histograms and skewness.

Since the normality assumption was violated, square root transformation was applied to the dependent variable for all groups.

Data transformation did not rectify the normality assumption violation for the SDS at pre-test, *W* (539) = 0.96, *p* ≤ 0.001; post-test, *W* (539) = 0.97, *p* ≤ 0.001; or follow-up, *W* (539) = 0.98, *p* ≤ 0.001. Results of the two-way mixed analysis of variances (ANOVA) on the original SDS data are reported as ANOVAs are robust to violations of normality assumptions (Lakens, 2022).

Data transformation rectified the violation for the AQ at pre-test, *W* (539) = 0.10, *p* = 0.975; post-test, *W* (539) = 0.10, *p* = 0.149; and follow-up, *W* (539) = 0.10, *p* = 0.334. Results of the two-way mixed ANOVA on the transformed AQ data are thus reported.

Levene’s tests (SOM Figure 4) found the homogeneity of variances assumption was violated for both measures. For the SDS, equal variances were indicated at follow-up, *F* (3, 535) = 0.904, *p* = 0.439, but unequal variances at pre-test, *F* (3, 535) = 4.04, *p* = 0.007 and post-test, F(3, 535) = 6.16, p =  < 0.001. For the AQ, equal variances were indicated at pre-test, *F* (3, 535) = 1.18, *p* = 0.137, and at follow-up, *F* (3, 535) = 2.63, *p* = 0.049; but unequal variances at post-test, *F* (3, 535) = 3.56, *p* = 0.014. The results of the mixed ANOVAs are nonetheless reported as groups were similar sizes, which reduces the type I error rate (Lakens, 2022).

Mauchly’s test (SOM Figure 5) indicated the sphericity assumption was violated for the AQ, *χ*^2^(2) = 47.20, *p* ≤ 0.001 and SDS, *χ*2(2) = 22.01, *p* ≤ 0.001.

For each measure, a two-way mixed ANOVA was conducted with video condition (EV, CV, CombV, CtrlV) and time (pre-test, post-test, follow-up) as between- and within-subject factors respectively.

Where a significant interaction effect was present, simple main effects through pairwise comparisons were calculated to explore how each group was differentially effective at each level of time. A Greenhouse Geisser correction was used for multiple comparisons.

Five-point Likert feasibility questions were analysed based on the percentage of participants scoring ‘agree’ (4) or ‘strongly agree’ (5). Three-point Likert questions were analysed through percentage of participants selecting each score. Qualitative responses were too short for comprehensive analysis but reviewed to identify frequently occurring themes.

## Results

### Participants

Six hundred and thirty-nine participants were recruited from May to June 2022. Six hundred met eligibility criteria, completed stage one and passed attention checks—150 in each group (EV, CV, CombV, CtrlV). Demonstrating a 90% retention rate, 539 participants completed stage two in July 2022 (Fig. 1), which was a sufficient sample (power, 1-*β* err prob, 1.00). Table 1 provides demographic information, which was broadly comparable across groups.

**Table 1:** Participant demographic information

	CtrlV	CombV	CV	EV	All
*N* =	*132*	*134*	*138*	*135*	*539*
Age					
18–24	8%	17%	12%	10%	12%
25–34	26%	28%	25%	26%	26%
35–44	25%	20%	25%	28%	25%
45–54	22%	16%	19%	20%	19%
55–64	12%	10%	12%	11%	11%
65–74	5%	7%	5%	3%	5%
75 +	1%	1%	1%	1%	1%
Prefer not to answer	1%	1%	1%	1%	1%
Gender					
Female	72%	70%	59%	49%	62%
Male	26%	28%	41%	50%	36%
Non-binary	2%	1%	1%	1%	1%
Prefer not to answer	0%	1%	0%	1%	0%
Ethnicity					
White	82%	81%	86%	78%	82%
Asian	8%	7%	4%	12%	8%
Mixed	4%	4%	4%	4%	4%
Black	5%	1%	4%	1%	3%
Other	1%	3%	1%	1%	2%
Prefer not to answer	1%	3%	1%	3%	2%
Personal experience (self)					
Yes—currently	2%	1%	4%	0%	2%
Yes—in the past	4%	7%	7%	13%	8%
No	95%	90%	88%	86%	89%
Prefer not to answer	0%	3%	1%	1%	1%
Personal experience (of others)					
Yes—currently	22%	21%	22%	17%	20%
Yes—in the past	45%	51%	43%	47%	47%
No	31%	26%	35%	34%	32%
Prefer not to answer	2%	1%	1%	1%	1%

### Intervention Effects

#### Stigma Scores Significantly Reduced Across Intervention Groups vs. Pre-test at Both Post-test and Follow-up Despite Significantly Increasing Between Post-test and Follow-up

AQ stigma (Table 2) significantly reduced for intervention groups between pre-test and both post-test (EV, MD = 1.18, SE = 0.07, *p* ≤ 0.001; CV, MD = 1.01, SE = 0.07, *p* ≤ 0.001; CombV, MD = 1.31, SE = 0.07, *p* ≤ 0.001) and follow-up (EV, MD = 0.66. SE = 0.09, *p* ≤ 0.001; CV, MD = 0.37, SE = 0.08, *p* ≤ 0.001; CombV, MD = 0.69, SE = 0.08, *p* ≤ 0.001) despite significantly increasing between post-test and follow-up (EV, MD =  − 0.52, SE = 0.09, *p* ≤ 0.001; CV, MD =  − 0.64, SE = 0.08, *p* ≤ 0.001; CombV, MD =  − 0.62, SE = 0.09, *p* ≤ 0.001).

**Table 2:** Pairwise comparisons for time (AQTr)

Pairwise comparisons							
Measure: AQTr							
Group	(I) Time	(J) Time	Mean difference (I–J)	Std. error	Sig.^b^	95% Confidence interval for difference^b^	
						Lower bound	Upper bound
CtrlV	1	2	.216^*^	.067	.001	.084	.348
		3	.270^*^	.085	.002	.102	.438
	2	1	− .216^*^	.067	.001	− .348	− .084
		3	.054	.086	.530	− .114	.222
	3	1	− .270^*^	.085	.002	− .438	− .102
		2	-.054	.086	.530	− .222	.114
EV	1	2	1.181^*^	.067	< .001	1.050	1.311
		3	.660^*^	.085	< .001	.493	.826
	2	1	− 1.181^*^	.067	< .001	− 1.311	− 1.050
		3	− .521^*^	.085	< .001	− .687	− .355
	3	1	− .660^*^	.085	< .001	− .826	− .493
		2	.521^*^	.085	< .001	.355	.687
CV	1	2	1.010^*^	.066	< .001	.880	1.139
		3	.370^*^	.084	< .001	.205	.534
	2	1	− 1.010^*^	.066	< .001	− 1.139	− .880
		3	− .640^*^	.084	< .001	− .804	− .476
	3	1	− .370^*^	.084	< .001	− .534	− .205
		2	.640^*^	.084	< .001	.476	.804
CombV	1	2	1.309^*^	.067	< .001	1.178	1.440
		3	.694^*^	.085	< .001	.527	.861
	2	1	− 1.309^*^	.067	< .001	− 1.440	− 1.178
		3	− .615^*^	.085	< .001	− .782	− .448
	3	1	− .694^*^	.085	< .001	− .861	− .527
		2	.615^*^	.085	< .001	.448	.782

Based on estimated marginal means

^*^The mean difference is significant at the .05 level

^b^Adjustment for multiple comparisons: least significant difference (equivalent to no adjustments)

SDS stigma (Table 3) significantly reduced for intervention groups between pre-test and both post-test (EV, MD = 0.36, SE = 0.03, *p* ≤ 0.001; CV, MD = 0.34, SE = 0.03, *p* ≤ 0.001; CombV, MD = 0.47, SE = 0.03, *p* ≤ 0.001) and follow-up (EV, MD = 0.24, SE = 0.03, *p* ≤ 0.001; CV, MD = 0.24, SE = 0.03, *p* ≤ 0.001; CombV, MD = 0.32, SE = 0.03, *p* ≤ 0.001) despite significantly increasing between post-test and follow-up (EV, MD =  − 0.12, SE = 0.04, *p* = 0.002; CV, MD = -0.10, SE = 0.04, *p* = 0.007; MD =  − 0.15, SE = 0.04, *p* ≤ 0.001).

**Table 3:** Pairwise comparisons for time (SDS)

Pairwise comparisons							
Measure: SDS							
Group	(I) Time	(J) Time	Mean difference (I–J)	Std. error	Sig.^b^	95% Confidence interval for difference^b^	
						Lower bound	Upper bound
CtrlV	1	2	.009	.033	.792	− .056	.073
		3	.137^*^	.034	< .001	.070	.205
	2	1	− .009	.033	.792	− .073	.056
		3	.129^*^	.039	< .001	.053	.205
	3	1	− .137^*^	.034	< .001	− .205	− .070
		2	− .129^*^	.039	< .001	− .205	− .053
EV	1	2	.364^*^	.032	< .001	.300	.428
		3	.244^*^	.034	< .001	.178	.311
	2	1	− .364^*^	.032	< .001	− .428	− .300
		3	− .120^*^	.038	.002	− .195	− .045
	3	1	− .244^*^	.034	< .001	− .311	− .178
		2	.120^*^	.038	.002	.045	.195
CV	1	2	.344^*^	.032	< .001	.281	.407
		3	.242^*^	.034	< .001	.176	.308
	2	1	− .344^*^	.032	< .001	− .407	− .281
		3	− .101^*^	.038	.007	− .176	− .027
	3	1	− .242^*^	.034	< .001	− .308	− .176
		2	.101^*^	.038	.007	.027	.176
CombV	1	2	.470^*^	.033	< .001	.406	.534
		3	.318^*^	.034	< .001	.251	.384
	2	1	− .470^*^	.033	< .001	− .534	− .406
		3	− .152^*^	.038	< .001	− .228	− .077
	3	1	− .318^*^	.034	< .001	− .384	− .251
		2	.152^*^	.038	< .001	.077	.228

Based on estimated marginal means

^*^The mean difference is significant at the .05 level

^b^Adjustment for multiple comparisons: least significant difference (equivalent to no adjustments)

AQ and SDS stigma for the CtrlV significantly reduced between pre-test and follow-up (AQ: MD = 0.27, SE = 0.09, *p* = 0.002; SDS: MD = 0.14, SE = 0.03, *p* ≤ 0.001). AQ stigma significantly reduced between pre- and post-test (MD = 0.22, SE = 0.07, *p* ≤ 0.001) but not SDS stigma (*p* > 0.05), whereas SDS stigma significantly reduced between post-test and follow-up (MD = 0.13, SE = 0.04, *p* ≤ 0.001) but not AQ stigma (MD = 0.05, SE = 0.09, *p* = 0.530).

#### Significant time-Group Interaction Effect Observed: Intervention Groups Displayed Significantly Reduced Stigma Relative to the Control Group at Post-assessment and Follow-up

A significant time-group interaction effect was observed for the AQ, *F* (5.53, 986.54) = 20.59, *p* < 0.001, and the SDS, *F* (5.77, 1028.47) = 16.67, *p* < 0.001, both with a medium effect size (partial *η*2 = 0.10, partial *η*2 = 0.085 respectively), meaning the study’s hypothesis (1) that there would be an interaction effect between time and group was supported.

Pairwise comparisons by group are in Table 4 (AQ) and Table 5 (SDS).

**Table 4:** Pairwise comparisons for group (AQTr)

Pairwise comparisons							
Measure: AQTr							
Time	(I) Group	(J) Group	Mean difference (I–J)	Std. error	Sig.^b^	95% Confidence interval for difference^b^	
						Lower bound	Upper bound
1	CtrlV	EV	.005	.160	.976	− .310	.320
		CV	.428^*^	.159	.007	.115	.741
		CombV	.150	.161	.351	− .165	.466
	EV	CtrlV	− .005	.160	.976	− .320	.310
		CV	.423^*^	.159	.008	.112	.735
		CombV	.145	.160	.364	− .169	.459
	CV	CtrlV	− .428^*^	.159	.007	− .741	− .115
		EV	− .423^*^	.159	.008	− .735	− .112
		CombV	− .278	.159	.081	− .590	.034
	CombV	CtrlV	− .150	.161	.351	− .466	.165
		EV	− .145	.160	.364	− .459	.169
		CV	.278	.159	.081	− .034	.590
2	CtrlV	EV	.969^*^	.181	< .001	.613	1.326
		CV	1.222^*^	.180	< .001	.867	1.576
		CombV	1.243^*^	.182	< .001	.886	1.600
	EV	CtrlV	− .969^*^	.181	< .001	− 1.326	− .613
		CV	.252	.179	.160	− .100	.605
		CombV	.274	.181	.131	− .081	.629
	CV	CtrlV	− 1.222^*^	.180	< .001	− 1.576	− .867
		EV	− .252	.179	.160	− .605	.100
		CombV	.021	.180	.906	− .332	.374
	CombV	CtrlV	− 1.243^*^	.182	< .001	− 1.600	− .886
		EV	− .274	.181	.131	− .629	.081
		CV	− .021	.180	.906	− .374	.332
3	CtrlV	EV	.395^*^	.179	.028	.042	.747
		CV	.528^*^	.178	.003	.178	.878
		CombV	.574^*^	.180	.001	.221	.927
	EV	CtrlV	− .395^*^	.179	.028	− .747	− .042
		CV	.133	.177	.452	− .215	.482
		CombV	.179	.179	.316	− .172	.530
	CV	CtrlV	− .528^*^	.178	.003	− .878	− .178
		EV	− .133	.177	.452	− .482	.215
		CombV	.046	.178	.796	− .303	.395
	CombV	CtrlV	− .574^*^	.180	.001	− .927	− .221
		EV	− .179	.179	.316	− .530	.172
		CV	− .046	.178	.796	− .395	.303

Based on estimated marginal means

^*^The mean difference is significant at the .05 level

^b^Adjustment for multiple comparisons: least significant difference (equivalent to no adjustments)

**Table 5:** Pairwise comparisons for group (SDS)

Pairwise comparisons							
Measure: SDS							
Time	(I) Group	(J) Group	Mean difference (I–J)	Std. error	Sig.^b^	95% Confidence interval for difference^b^	
						Lower bound	Upper bound
1	CtrlV	EV	.094	.050	.061	− .004	.193
		CV	.073	.050	.146	− .025	.170
		CombV	.008	.050	.870	− .090	.107
	EV	CtrlV	− .094	.050	.061	− .193	.004
		CV	− .022	.050	.662	− .119	.076
		CombV	− .086	.050	.085	− .184	.012
	CV	CtrlV	− .073	.050	.146	− .170	.025
		EV	.022	.050	.662	− .076	.119
		CombV	− .064	.050	.196	− .162	.033
	CombV	CtrlV	− .008	.050	.870	− .107	.090
		EV	.086	.050	.085	− .012	.184
		CV	.064	.050	.196	− .033	.162
2	CtrlV	EV	.450^*^	.065	< .001	.322	.577
		CV	.408^*^	.065	< .001	.280	.535
		CombV	.470^*^	.065	< .001	.342	.598
	EV	CtrlV	− .450^*^	.065	< .001	− .577	− .322
		CV	− .042	.064	.514	− .168	.084
		CombV	.020	.065	.757	− .107	.148
	CV	CtrlV	− .408^*^	.065	< .001	− .535	− .280
		EV	.042	.064	.514	− .084	.168
		CombV	.062	.065	.336	− .065	.189
	CombV	CtrlV	− .470^*^	.065	< .001	− .598	− .342
		EV	− .020	.065	.757	− .148	.107
		CV	− .062	.065	.336	− .189	.065
3	CtrlV	EV	.201^*^	.062	.001	.080	.323
		CV	.177^*^	.062	.004	.056	.298
		CombV	.188^*^	.062	.003	.067	.310
	EV	CtrlV	− .201^*^	.062	.001	− .323	− .080
		CV	− .024	.061	.697	− .144	.096
		CombV	− .013	.062	.836	− .134	.108
	CV	CtrlV	− .177^*^	.062	.004	− .298	− .056
		EV	.024	.061	.697	− .096	.144
		CombV	.011	.061	.856	− .109	.132
	CombV	CtrlV	− .188^*^	.062	.003	− .310	− .067
		EV	.013	.062	.836	− .108	.134
		CV	− .011	.061	.856	− .132	.109

Based on estimated marginal means

^*^The mean difference is significant at the .05 level

^b^Adjustment for multiple comparisons: least significant difference (equivalent to no adjustments)

While mean AQ stigma in the CtrlV significantly reduced at post-test, mean AQ and SDS stigma was significantly lower in the intervention groups at post-test: EV (AQ: MD =  − 0.97, SE = 0.18, *p* ≤ 0.001; SDS: MD =  − 0.45, SE = 0.07, *p* ≤ 0.001), CV (AQ: MD =  − 1.22, SE = 0.18, *p* ≤ 0.001; SDS: MD =  − 0.41, SE = 0.07, *p* ≤ 0.001) and CombV (AQ: MD =  − 1.24, SE = 0.18, *p* ≤ 0.001; SDS: MD =  − 0.47, SE = 0.07, *p* ≤ 0.001). Stigma reductions at that time were similar across intervention groups despite marginal differences (all *p*s > 0.13).

While mean stigma in the CtrlV continued decreasing at follow-up, mean AQ and SDS stigma was still significantly lower in the intervention groups: EV (AQ: MD =  − 0.40, SE = 0.18, *p* = 0.028; SDS: MD =  − 0.20, SE = 0.06, *p* ≤ 0.001), CV (AQ: MD =  − 0.53, SE = 0.18, *p* = 0.003; SDS: MD =  − 0.18, SE = 0.06, *p* = 0.004) and CombV (AQ: MD =  − 0.57, SE = 0.18, *p* = 0.001; SDS: MD =  − 0.19, SE = 0.06, *p* = 0.003).

Consequently, the study’s hypothesis (2) that there would be a significant difference in the intervention groups’ mean stigma scores across time versus the CtrlV was supported. Again, there were no significant differences in mean AQ and SDS stigma between intervention groups (all *p*s > 0.31). For example, stigma in the CombV was lower but not significantly lower than the CV (AQ: MD =  − 0.05, SE = 0.18, *p* = 0.796; SDS: MD =  − 0.01, SE = 0.06, *p* = 0.86).

As differences between time points and intervention groups were not consistent, as shown by Fig. 2a (AQ) and Fig. 2b (SDS), neither a main effect of time nor group on mean stigma appears present.

### Feasibility

Feasibility of the study’s procedures and interventions (Tables 6 and 7) is summarised below against Orsmond and Cohn’s (2015) evaluation areas for feasibility research.

**Table 6:** Feasibility quantitative responses

	CtrlV			CombV			CV			EV		
	*% agree*	*% neither agree nor disagree*	*% disagree*	*% agree*	*% neither agree nor disagree*	*% disagree*	*% agree*	*% neither agree nor disagree*	*% disagree*	*% agree*	*% neither agree nor disagree*	*% disagree*
The initial information sheet gave me a clear explanation of what taking part in the study would involve	95%	4%	1%	96%	3%	1%	99%	0%	1%	98%	1%	1%
The initial instructions and briefing on the questionnaires meant I understood what was being asked of me	99%	1%	0%	99%	1%	1%	99%	0%	1%	98%	1%	1%
The questions and statements on the questionnaires were clear and easy to understand	96%	4%	0%	99%	0%	1%	99%	1%	0%	99%	1%	1%
I was able to accurately record my feelings towards John using the scoring scale provided on the questionnaires	95%	3%	2%	96%	2%	2%	95%	5%	0%	93%	3%	4%
	*Too short*	*About right*	*Too Long*	*Too short*	*About right*	*Too Long*	*Too short*	*About right*	*Too Long*	*Too short*	*About right*	*Too Long*
The time it took me to fill out the questionnaires was…	1%	95%	4%	0%	92%	8%	0%	99%	1%	0%	96%	4%
	*% Agree*	*% neither agree nor disagree*	*% disagree*	*% Agree*	*% neither agree nor disagree*	*% disagree*	*% Agree*	*% neither agree nor disagree*	*% disagree*	*% Agree*	*% neither agree nor disagree*	*% disagree*
The video was clear and easy to follow	95%	4%	1%	98%	1%	1%	100%	0%	0%	100%	0%	0%
The content in the video was engaging and interesting	85%	11%	4%	88%	8%	4%	95%	5%	0%	82%	12%	6%
The content in the video felt relevant to me	25%	32%	43%	50%	32%	18%	50%	32%	17%	60%	23%	17%
I learned information that I didn’t previously know	89%	8%	3%	76%	10%	14%	50%	21%	29%	76%	11%	13%
I would choose to watch this video in full in my own time	34%	25%	41%	48%	23%	30%	41%	26%	33%	46%	27%	27%
	*Too short*	*About right*	*Too Long*	*Too short*	*About right*	*Too Long*	*Too short*	*About right*	*Too Long*	*Too short*	*About right*	*Too Long*
The video length was…	1%	78%	21%	1%	66%	33%	1%	94%	5%	1%	84%	16%

**Table 7:** Feasibility selected qualitative responses

	Key themes	Example feedback
Education		
*Strengths*	Clear	*‘Clear explanations of myths and facts’; ‘It was clear and concise’; ‘The points were made clearly’; ‘Very clear illustrative statements’*
	Easy to follow	*‘Easy and simple to follow’; ‘It was laid out with an easy to follow structure’; ‘Simple straightforward presentation’; ‘The infographics helped me absorb the information’*
	Informative	*‘Lots of new information’; ‘Gave me new insight’; ‘I learnt a lot that I previously didn’t know’; 'I like the emphasis on clearing up myths with data-backed facts'*
*Improvements*	Production quality	*‘More variation in tone of voice’; ‘Speed up the narration’; ‘More colour and animation’*
	Additional content	*‘More statistics’; ‘Evidence for some of the facts’; ‘Where people could look for more info/support’; ‘Some real quotes’; ‘Add personal experiences from real people’*
	Shorter	*‘It was a bit long’; ‘Slightly shorter’; ‘Make it a little shorter’*
Contact		
*Strengths*	Speakers’ qualities	*‘Approachable, open and relatable speakers’; ‘The people seemed sincere and were engaging’; ‘I liked the honesty and braveness of the speakers’; ‘[They] felt genuine’*
	Relatable	*‘It was personal and relatable’; 'Real people sharing their real experiences'; ‘The people seemed like my friends and neighbours’; ‘I liked that they were just ordinary people’; ‘I liked how they humanised the experience’; ‘It made me empathise’*
	Diverse perspectives	*‘3 different people’s experiences…showed different perspectives’; ‘they seemed very different….showing that [AUD] affects lots of different types of people’; ‘I liked that the video featured people from different backgrounds including race and gender’*
	Balanced narrative	*‘I like that it followed a path’; ‘It told me how they got addicted’; ‘I liked the fact that [they] have found recovery and now live happier and more fulfilled lives’; ‘I liked how they also added difficulties they still currently face’; ‘Both positives and negatives of their journeys’*
*Improvements*	Production quality	*‘Could be more visually engaging’; ‘More engaging background, see a bit more of their home, life or workplace’; ‘Higher camera quality’*
	Additional content	*‘Include helplines’; ‘More on how to support people with addiction’; ‘A few more facts and figures’; ‘Hearing from the friends and family’; ‘Something from their…partners’;’Perspectives from health experts’; ‘More stories as 3 seemed too few’*
	Shorter	*‘Maybe slightly shorter’; ‘A little bit shorter’; ‘The video was too long’*
Combined		
*Strengths*	Personal stories	*‘I liked the personal aspect of it with the 3 people who gave their stories’; ‘The interviews made it more personal and engaging’; ‘Real life scenarios helps to place [AUD] in a context’*
	Educational	*'I liked the concise information about alcoholism and debunking of the myths’; ‘I liked learning about the myths of alcoholism’; ‘The myths and facts were interesting’*
	Combination of approaches	*'I liked that there were myths and facts but then you also heard from 3 different people that had dealt with alcoholism'; ‘The video was emotional, yet logical’; ‘It was informative and included real people’s stories’*
*Improvements*	Production quality	*‘The myth/facts section could be a tiny bit snappier’; ‘The commentary for the beginning part could be quicker’; ‘Sound quality could slightly improve’*
	Additional content	*‘Places to get help’; ‘Provide useful resources’; ‘How other people can push the government to do more’; ‘More real stories’; ‘Maybe [show] a variety of ages’; ‘[Show] people who are going through the journey but not through yet’*
	Shorter	*‘Would be better to reduce the time so people don’t lose interest’*
	Realistic information	*‘I felt it tilted towards too positive’; ‘More honesty about the chances of staying sober’; ‘Some [people with AUD] are violent’; ‘Accept people have a responsibility for their own actions too’*

#### Recruitment Capability and Resulting Sample Characteristics

Recruitment capability was strong and eligibility criteria feasible, with 632 eligible members of the target population recruited within a week (~ 2 days per group). Scores on pre-test stigma measures showed sufficiently high stigma scores to warrant intervention. It was, however, challenging to recruit a representative sample, with a skew towards younger (63% under 45 vs. 42% for UK population), female (64% vs. 52%) participants and those with personal experience of AUD (10% vs. 3%) (McManus et al., 2016).

#### Data Collection Procedures and Outcome Measures

Supporting the measures’ suitability for the intended population, participants felt instructions outlined what was expected (99% agreement), items were clear and easy to understand (98%) and they could accurately record feelings towards the vignette subject (95%). Indicators of internal consistency were good (e.g., alpha reliability coefficients above 0.80), though the measures’ appropriateness merits further research as they lack wide validation for measuring public AUD stigma. Usable and complete data were collected from all participants, with 92% also providing qualitative feasibility comments.

#### Acceptability and Suitability of Study Procedures and Intervention

Adherence and retention were strong, with 96% of participants completing stage one and a 90% retention rate at follow-up. Participants appeared to understand and engage with the interventions: most passed attention check questions (99%) and agreed the videos were clear and easy to follow (98–100%) and engaging and interesting (82–95%). Average study completion time was below 20 min and most participants felt survey duration was ‘about right’ (92–99%).

Qualitative feedback reflected participants’ satisfaction with the interventions. Each approach had different benefits, with those watching myth-fact content feeling they learned more new information (EV and CombV: 76% vs. CV: 50%) and contact content promoting empathy. Other strengths were the EV’s clarity and simplicity, the CV’s speakers, diversity and narrative, and CombV’s combined approaches.

The interventions’ acceptability was limited by their perceived relevance to participants (50–60%) and participants’ willingness to watch them in their own time (41–48%). Additionally, while most felt interventions were the right length (66–94%), qualitative feedback (especially for the CombV) recommended shortening them. Development areas included enhancing production quality, providing additional content and offering more balanced information.

#### Resources and Ability to Manage and Implement the Study and Intervention

The research team had the resources, time and skills to conduct the study. However, feasibility was limited by budgetary constraints for intervention production, where additional funding could have supported specialist input to increase intervention quality.

#### Participant Responses to Intervention

Participants’ stigma scores suggested all interventions show promise of being successful with the public (Fig. 2). Qualitative feedback also indicated their potential for attitude change (‘it changed my opinion about the condition’—CombV).

## Discussion

### Summary of Findings and Implications

The present study is, to our knowledge, the first to examine the relative efficacy of education and contact interventions in reducing the public stigma of AUD. Supporting the study’s hypothesis (H1), there was a significant interaction between time and group. Stigma in the intervention groups significantly decreased at post-test and follow-up, despite a significant rebound between post-test and follow-up. Supporting hypothesis (H2), while stigma scores decreased in the control group (CtrlV) between pre-test and follow-up, they decreased more in the intervention groups (EV, CV, CombV). Together, these findings suggest that brief education, contact and combined interventions used to tackle the public stigma of various neuropsychiatric disorders can successfully reduce public AUD stigma. Diminished intervention impact at follow-up is consistent with previous reports (Morgan et al., 2018), raising a potential challenge with using short interventions to sustainably shift entrenched social biases. Conceptual models of stigma highlight its perceived functions such as to keep outgroups ‘down’ (e.g., exploitation or domination) or ‘away’ (e.g., avoidance of illness). In the case of substance use disorders, it is often used to ‘keep people in’ by enforcing social norms (Phelan et al., 2008). The rigidity of public AUD stigma relative to other conditions (Crisp et al., 2005; Kilian et al., 2021; Schomerus et al., 2014) may, therefore, be explained by it being seen as an effective strategy to limit excessive alcohol use (Vanyukov, 2024) by defining the boundaries of acceptable behaviour (Phelan et al., 2008). Additionally, the interventions’ reduced impact at follow-up could reflect people’s tendency to avoid ‘cognitive dissonance’ (i.e., holding contradictory beliefs) through retaining their existing beliefs despite new information (Festinger, 1957). Further research is needed to clarify reasons for the interventions’ decreased impact over time and to create more sustained changes in stigma.

The contact content’s efficacy corroborates previous demonstration that positive portrayals of people with substance use disorder promote stigma reduction (Luty et al., 2009; McGinty et al., 2015). Participants’ qualitative endorsement of its diverse speakers, and balancing of symptoms, recovery and ongoing difficulties, also substantiate anti-stigma recommendations for contact intervention development (Corrigan et al., 2013). However, contrary to evidence that education is either ineffective for the reduction of AUD stigma (Luty et al., 2007) or inferior to contact (Corrigan et al., 2007), this study found no significant differences in efficacy between the two types of intervention. Combining both approaches in anti-stigma efforts may, therefore, be useful, especially given participants highlighted their different benefits, with education imparting knowledge and contact humanising people with AUD.

More research is needed to understand these discrepancies. However, future trials may consider engaging mediums for education content delivery since our myths and facts were effective in an animated explainer format, in contrast to previously ineffective black-and-white factsheets (Luty et al., 2007). Additionally, systematic content selection may enhance intervention effects: our myth-fact pairs were developed based on expert perspectives and a systematic review of population studies of public attitudes towards AUD, ensuring messaging reflected public attitudes and key AUD stereotypes.

Our findings support progression to a larger trial, with some amendments to the study’s procedures and interventions. While a large sample was enrolled in a short period, a more representative sample is needed to confirm the interventions’ public impact. Data collection procedures enabled sufficient data analyses, and the outcome measures were reliable and perceived as appropriate. However, further validation of measures examining the public stigma of addictive disorders (Brown, 2011; Johnson-Kwochka et al., 2021) would enable more accurate future evaluations. Study procedures were considered suitable, and most participants were satisfied with the study duration and retained at follow-up. Further, the interventions show promise for stigma reduction in future trials, given both their effects and qualitative feedback about their potential for attitude change. However, while their acceptability was endorsed, more work is needed to improve their relevance and appeal to participants since less than half agreed they would watch the videos in their own time. Feedback suggested future studies would benefit from shortening the videos, enhancing their quality (particularly making education content more engaging) and providing additional content (e.g., support resources). Offering a more balanced perspective (e.g., realistic recovery rates) was also advised, suggesting holistic accounts of AUD are important while countering stigma. Greater resources to improve the interventions prior to further testing may result in an approach that is more likely to succeed.

## Limitations

This study’s findings should be considered in the context of its limitations. First, the sample, recruited through the online crowd-sourcing platform Prolific, was not entirely representative of the UK public (Chandler et al., 2015), limiting the generalisability of the study’s findings. It was skewed towards younger, female participants and those with direct and indirect experience of AUD. While gender did not materially impact stigma, younger participants and those with experience of AUD demonstrated slightly lower stigma across time.

Second, the comparability of effects across groups was limited because the study was not truly randomised since participants entered it at different times. Additionally, the difference in length of the CtrlV and CombV makes the latter’s efficacy challenging to assess quantitatively.

Third, the findings lack ecological validity. Measures assessed explicit rather than implicit attitudes (Bos et al., 2013), which have been shown to reduce more following anti-stigma interventions (Maunder & White, 2019). Whether self-reported behavioural intentions would translate to actual behaviour is therefore unclear. Further, reduced intervention impact at follow-up and changes in control group stigma suggest social desirability bias may have influenced results.

Finally, while 1 month is a longer follow-up than other contact and education studies (Corrigan et al., 2002, 2004, 2007), establishing the duration of intervention effects on stigma over longer periods is important given increased stigma at follow-up in our study.

## Directions for Future Research

To verify these preliminary results, a future larger trial could capitalise on the insights offered by the present study and improve their generalisability (e.g., through true randomisation or a more representative sample) and feasibility (e.g., through better-produced interventions). To improve ecological validity, the interventions could be tested in real-life contexts, with changes in actual behaviour evaluated (e.g., donations to charity). They could be trialled initially in sub-groups of the population (e.g., students), with the aim of broadening their use in society-wide campaigns (e.g., via social media or short public health adverts). Alternatively, developing targeted interventions for specific groups able to effect change, such as policymakers or employers, may be impactful (Corrigan et al., 2014).

Since public stigma of AUD is entrenched (Crisp et al., 2005; Kilian et al., 2021; Nathan et al., 2016), it is possible that single-session interventions promote insufficient lasting change (Earnshaw et al., 2018). Future research could, therefore, evaluate the impact of the interventions when delivered continuously. Similarly, research into intervention acceptability could elucidate how to prolong effects at follow-up.

Additionally, studies into mechanisms of change could clarify how the interventions are efficacious when applied to addiction-related stigma. For example, while the contact hypothesis proposes enhanced knowledge as the key mediator of contact interventions (Allport, 1954), contemporary research regards other mediators, such as empathy, as superior (Pettigrew & Tropp, 2008).

To facilitate engagement, additional studies could test the effects of interventions delivered at different lengths (e.g., 4 versus 2 min), through different mediums (e.g., text versus video) and by different people (e.g., actors, celebrities, health professionals). Further, other education and contact intervention types could be tested, such as facts-only (Luty et al., 2007) and vignette contact interventions (Luty et al., 2008).

Finally, interventions found to be successful after more extensive evaluation should be tailored to other substance and behavioural addictions in order not only to determine the differential sensitivity of stigmas towards these conditions to these interventions but also to develop a wide range of strategies effective at reducing public stigma towards all addictions.

## Supplementary Information

Below is the link to the electronic supplementary material.

Supplementary file1 (PDF 1441 KB)

## Data Availability

Data will be available on request.

## References

[CR1] Abdullah, T., & Brown, T. L. (2020). Diagnostic labeling and mental illness stigma among Black Americans: An experimental vignette study. *Stigma and Health,**5*(1), 11–21. 10.1037/sah0000162

[CR2] Abraham, A. J., Bride, B. E., & Roman, P. M. (2013). Public attitudes toward persons with alcohol use disorders (AUDs): The role of social contact and treatment-seeking behavior. *Sociological Focus,**46*(4), 267–280. 10.1080/00380237.2013.825542

[CR3] Allport, G. W. (1954). The nature of prejudice. *Addison-Wesley Google Schola,**2*, 59–82.

[CR4] Allport, G. W., Clark, K., & Pettigrew, T. (1979). *The nature of prejudice* (25th anniversary ed.). Basic Books.

[CR5] American Psychiatric Association. (2000). *Diagnostic and statistical manual of mental disorders Text revision (DSM-IV TR)* (4th ed.).

[CR6] Berney, S., & Bétrancourt, M. (2016). Does animation enhance learning? A meta-analysis. *Computers & Education,**101*, 150–167. 10.1016/j.compedu.2016.06.005

[CR7] Bos, A. E. R., Pryor, J. B., Reeder, G. D., & Stutterheim, S. E. (2013). Stigma: Advances in theory and research. *Basic and Applied Social Psychology,**35*(1), 1–9. 10.1080/01973533.2012.746147

[CR8] Brown, S. A. (2008). Factors and measurement of mental illness stigma: A psychometric examination of the Attribution Questionnaire. *Psychiatric Rehabilitation Journal,**32*(2), 89–94. 10.2975/32.2.2008.89.94 18840562 10.2975/32.2.2008.89.94

[CR9] Brown, S. A. (2011). Standardized measures for substance use stigma. *Drug and Alcohol Dependence,**116*(1), 137–141. 10.1016/j.drugalcdep.2010.12.005 21257274 10.1016/j.drugalcdep.2010.12.005

[CR10] Chan, J. Y. N., Mak, W. W. S., & Law, L. S. C. (2009). Combining education and video-based contact to reduce stigma of mental illness: “The Same or Not the Same” anti-stigma program for secondary schools in Hong Kong. *Social Science & Medicine,**68*(8), 1521–1526. 10.1016/j.socscimed.2009.02.016 19282079 10.1016/j.socscimed.2009.02.016

[CR11] Chandler, J., Paolacci, G., Peer, E., Mueller, P., & Ratliff, K. A. (2015). Using nonnaive participants can reduce effect sizes. *Psychological Science,**26*(7), 1131–1139. 10.1177/0956797615585115 26063440 10.1177/0956797615585115

[CR12] Cohen, J. (1988). *Statistical Power Analysis for the Behavioral Sciences* (2nd ed.). Routledge. 10.4324/9780203771587

[CR13] Corrigan, P. W. (2015). Challenging the Stigma of Mental Illness: Different Agendas: Different Goals. *Psychiatric Services,**66*(12), 1347–1349. 10.1176/appi.ps.201500107 26278234 10.1176/appi.ps.201500107

[CR14] Corrigan, P. W., & Shapiro, J. R. (2010). Measuring the impact of programs that challenge the public stigma of mental illness. *Clinical Psychology Review,**30*(8), 907–922. 10.1016/j.cpr.2010.06.004 20674114 10.1016/j.cpr.2010.06.004 PMC2952670

[CR15] Corrigan, P. W., River, L. P., Lundin, R. K., Penn, D. L., Uphoff-Wasowski, K., Campion, J., Mathisen, J., Gagnon, C., Bergman, M., Goldstein, H., & Kubiak, M. A. (2001). Three strategies for changing attributions about severe mental illness. *Schizophrenia Bulletin,**27*(2), 187–195. 10.1093/oxfordjournals.schbul.a006865 11354586 10.1093/oxfordjournals.schbul.a006865

[CR16] Corrigan, P. W., Rowan, D., Green, A., Lundin, R., River, P., Uphoff-Wasowski, K., White, K., & Kubiak, M. A. (2002). Challenging two mental illness stigmas: Personal responsibility and dangerousness. *Schizophrenia Bulletin,**28*(2), 293–309. 10.1093/oxfordjournals.schbul.a006939 12693435 10.1093/oxfordjournals.schbul.a006939

[CR17] Corrigan, P. W., Watson, A. C., Warpinski, A. C., & Gracia, G. (2004). Implications of educating the public on mental illness, violence, and stigma. *Psychiatric Services,**55*(5), 577–580. 10.1176/appi.ps.55.5.577 15128968 10.1176/appi.ps.55.5.577

[CR18] Corrigan, P. W., Larson, J., Sells, M., Niessen, N., & Watson, A. C. (2007). Will filmed presentations of education and contact diminish mental illness stigma? *Community Mental Health Journal,**43*(2), 171–181. 10.1007/s10597-006-9061-8 16988883 10.1007/s10597-006-9061-8

[CR19] Corrigan, P. W., Rafacz, J., & Rüsch, N. (2011). Examining a progressive model of self-stigma and its impact on people with serious mental illness. *Psychiatry Research,**189*(3), 339–343. 10.1016/j.psychres.2011.05.024 21715017 10.1016/j.psychres.2011.05.024 PMC3185170

[CR20] Corrigan, P. W., Morris, S. B., Michaels, P. J., Rafacz, J. D., & Rüsch, N. (2012). Challenging the public stigma of mental illness: A meta-analysis of outcome studies. *Psychiatric Services,**63*(10), 963–973. 10.1176/appi.ps.201100529 23032675 10.1176/appi.ps.201100529

[CR21] Corrigan, P. W., Vega, E., Larson, J., Michaels, P. J., McClintock, G., Krzyzanowski, R., Gause, M., & Buchholz, B. (2013). The California schedule of key ingredients for contact-based antistigma programs. *Psychiatric Rehabilitation Journal,**36*(3), 173–179. 10.1037/prj0000006 23834612 10.1037/prj0000006

[CR22] Corrigan, P. W., Michaels, P. J., Vega, E., Gause, M., Larson, J., Krzyzanowski, R., & Botcheva, L. (2014). Key ingredients to contact-based stigma change: A cross-validation. *Psychiatric Rehabilitation Journal,**37*(1), 62–64. 10.1037/prj0000038 24417232 10.1037/prj0000038

[CR23] Corrigan, P. W., Schomerus, G., Shuman, V., Kraus, D., Perlick, D., Harnish, A., Kulesza, M., Kane-Willis, K., Qin, S., & Smelson, D. (2017). Developing a research agenda for reducing the stigma of addictions, part II: Lessons from the mental health stigma literature. *The American Journal on Addictions,**26*(1), 67–74. 10.1111/ajad.12436 27875626 10.1111/ajad.12436

[CR24] Corrigan, P. (2008). A TOOLKIT for evaluating programs meant to erase the stigma of mental illness

[CR25] Craig, P., Dieppe, P., Macintyre, S., Michie, S., Nazareth, I., & Petticrew, M. (2008). Developing and evaluating complex interventions: The new Medical Research Council guidance. *BMJ,**337*, a1655. 10.1136/bmj.a1655 18824488 10.1136/bmj.a1655 PMC2769032

[CR26] Crapanzano, K., Hammarlund, R., Ahmad, B., Hunsinger, N., & Kullar, R. (2018). The association between perceived stigma and substance use disorder treatment outcomes: A review. *Substance Abuse and Rehabilitation,**10*, 1–12. 10.2147/sar.s183252 30643480 10.2147/SAR.S183252 PMC6311321

[CR27] Crisp, A., Gelder, M., Goddard, E., & Meltzer, H. (2005). Stigmatization of people with mental illnesses: A follow-up study within the Changing Minds campaign of the Royal College of Psychiatrists. *World Psychiatry*,* 4*(2), 106–113. https://www.ncbi.nlm.nih.gov/pubmed/16633526 PMC1414750 16633526

[CR28] Earnshaw, V. A., Reisner, S. L., Menino, D. D., Poteat, V. P., Bogart, L. M., Barnes, T. N., & Schuster, M. A. (2018). Stigma-based bullying interventions: A systematic review. *Developmental Review,**48*, 178–200. 10.1016/j.dr.2018.02.001 30220766 10.1016/j.dr.2018.02.001 PMC6135094

[CR29] Evans-Lacko, S., London, J., Japhet, S., Rüsch, N., Flach, C., Corker, E., Henderson, C., & Thornicroft, G. (2012). Mass social contact interventions and their effect on mental health related stigma and intended discrimination. *BMC Public Health,**12*(1), 489. 10.1186/1471-2458-12-489 22742085 10.1186/1471-2458-12-489 PMC3461459

[CR30] Festinger, L. (1957). *A theory of cognitive dissonance*. Stanford University Press.

[CR31] Finn, S. W., Mejldal, A., & Nielsen, A. S. (2023). Public stigma and treatment preferences for alcohol use disorders. *BMC Health Services Research,**23*(1), 76. 10.1186/s12913-023-09037-y 36694198 10.1186/s12913-023-09037-y PMC9872434

[CR32] Glass, J. E., Mowbray, O. P., Link, B. G., Kristjansson, S. D., & Bucholz, K. K. (2013). Alcohol stigma and persistence of alcohol and other psychiatric disorders: A modified labeling theory approach. *Drug and Alcohol Dependence,**133*(2), 685–692. 10.1016/j.drugalcdep.2013.08.016 24071569 10.1016/j.drugalcdep.2013.08.016 PMC3980578

[CR33] Goffman, E. (1963). *Stigma. Notes on the management of spoiled identity*. Penguin.

[CR34] Griffiths, K. M., Carron-Arthur, B., Parsons, A., & Reid, R. (2014). Effectiveness of programs for reducing the stigma associated with mental disorders. A meta-analysis of randomized controlled trials. *World Psychiatry,**13*(2), 161–175. 10.1002/wps.20129 24890069 10.1002/wps.20129 PMC4102289

[CR35] Höffler, T. N., & Leutner, D. (2007). Instructional animation versus static pictures: A meta-analysis. *Learning and Instruction,**17*(6), 722–738. 10.1016/j.learninstruc.2007.09.013

[CR36] https://www.ncbi.nlm.nih.gov/pmc/articles/PMC1414750/pdf/wpa040106.pdf

[CR37] Hunter, B. A., Mohatt, N. V., Prince, D. M., Thompson, A. B., Matlin, S. L., & Tebes, J. K. (2017). Socio-psychological mediators of the relationship between behavioral health stigma and psychiatric symptoms. *Social Science & Medicine,**181*, 177–183. 10.1016/j.socscimed.2017.03.049 28407602 10.1016/j.socscimed.2017.03.049 PMC6557155

[CR38] Janulis, P., Ferrari, J. R., & Fowler, P. (2013). Understanding public stigma toward substance dependence. *Journal of Applied Social Psychology,**43*(5), 1065–1072. 10.1111/jasp.12070

[CR39] Johnson-Kwochka, A., Aalsma, M. C., Monahan, P. O., & Salyers, M. P. (2021). Development and examination of the attribution questionnaire-substance use disorder (AQ-SUD) to measure public stigma towards adolescents experiencing substance use disorders. *Drug and Alcohol Dependence,**221*, 108600. 10.1016/j.drugalcdep.2021.108600 33689966 10.1016/j.drugalcdep.2021.108600

[CR40] Jones, E. E. (1984). *Social stigma: The psychology of marked relationships*. W.H Freeman.

[CR41] Keith, M. G., Tay, L., & Harms, P. D. (2017). Systems perspective of Amazon Mechanical Turk for organizational research: Review and recommendations [Review]. *Frontiers in Psychology,**8*, 1359. 10.3389/fpsyg.2017.01359 28848474 10.3389/fpsyg.2017.01359 PMC5550837

[CR42] Keyes, K. M., Hatzenbuehler, M. L., McLaughlin, K. A., Link, B., Olfson, M., Grant, B. F., & Hasin, D. (2010). Stigma and treatment for alcohol disorders in the United States. *American Journal of Epidemiology,**172*(12), 1364–1372. 10.1093/aje/kwq304 21044992 10.1093/aje/kwq304 PMC2998202

[CR43] Kilian, C., Manthey, J., Carr, S., Hanschmidt, F., Rehm, J., Speerforck, S., & Schomerus, G. (2021). Stigmatization of people with alcohol use disorders: An updated systematic review of population studies. *Alcohol: Clinical and Experimental Research,**45*(5), 899–911. 10.1111/acer.14598 10.1111/acer.14598 33970504

[CR44] Lakens, D. (2022). Improving your statistical inferences

[CR45] Link, B. G. (1987). Understanding labeling effects in the area of mental disorders: An assessment of the effects of expectations of rejection. *American Sociological Review,**52*(1), 96–112. 10.2307/2095395

[CR46] Link, B. G., & Phelan, J. C. (2001). Conceptualizing stigma. *Annual Review of Sociology,**27*(27), 363–385. 10.1146/annurev.soc.27.1.363

[CR47] Livingston, J. D., Milne, T., Fang, M. L., & Amari, E. (2012). The effectiveness of interventions for reducing stigma related to substance use disorders: A systematic review. *Addiction,**107*(1), 39–50. 10.1111/j.1360-0443.2011.03601.x 21815959 10.1111/j.1360-0443.2011.03601.x PMC3272222

[CR48] Luoma, J. B., Kulesza, M., Hayes, S. C., Kohlenberg, B., & Larimer, M. (2014). Stigma predicts residential treatment length for substance use disorder. *The American Journal of Drug and Alcohol Abuse,**40*(3), 206–212. 10.3109/00952990.2014.901337 24766087 10.3109/00952990.2014.901337 PMC5061110

[CR49] Luty, J., Umoh, O., Sessay, M., & Sarkhel, A. (2007). Effectiveness of Changing Minds campaign factsheets in reducing stigmatised attitudes towards mental illness. *Psychiatric Bulletin,**31*(10), 377–381. 10.1192/pb.bp.106.012443

[CR50] Luty, J., Rao, H., Arokiadass, S. M. R., Easow, J. M., & Sarkhel, A. (2008). The repentant sinner: Methods to reduce stigmatised attitudes towards mental illness. *Psychiatric Bulletin,**32*(9), 327–332. 10.1192/pb.bp.107.018457

[CR51] Luty, J., Umoh, O., & Nuamah, F. (2009). Effect of brief motivational interviewing on stigmatised attitudes towards mental illness. *Psychiatric Bulletin,**33*(6), 212–214. 10.1192/pb.bp.108.020925

[CR52] Mak, W. W., Chan, R. C., Wong, S. Y., Lau, J. T., Tang, W. K., Tang, A. K., Chiang, T. P., Cheng, S. K., Chan, F., Cheung, F. M., Woo, J., & Lee, D. T. (2017). A cross-diagnostic investigation of the differential impact of discrimination on clinical and personal recovery. *Psychiatric Services (Washington, d. c.),**68*(2), 159–166. 10.1176/appi.ps.201500339 27842474 10.1176/appi.ps.201500339

[CR53] Maunder, R. D., & White, F. A. (2019). Intergroup contact and mental health stigma: A comparative effectiveness meta-analysis. *Clinical Psychology Review,**72*, 101749. 10.1016/j.cpr.2019.101749 31254936 10.1016/j.cpr.2019.101749

[CR54] McGinty, E. E., Goldman, H. H., Pescosolido, B., & Barry, C. L. (2015). Portraying mental illness and drug addiction as treatable health conditions: Effects of a randomized experiment on stigma and discrimination. *Social Science & Medicine,**126*, 73–85. 10.1016/j.socscimed.2014.12.010 25528557 10.1016/j.socscimed.2014.12.010

[CR55] McManus, S., Bebbington, P., Jenkins, R., & Brugha, T. (2016). *Mental health and wellbeing in England: Adult Psychiatric Morbidity Survey 2014*

[CR56] Michie, S., van Stralen, M. M., & West, R. (2011). The behaviour change wheel: A new method for characterising and designing behaviour change interventions. *Implementation Science,**6*(1), 42. 10.1186/1748-5908-6-42 21513547 10.1186/1748-5908-6-42 PMC3096582

[CR57] Morgan, A. J., Reavley, N. J., Ross, A., Too, L. S., & Jorm, A. F. (2018). Interventions to reduce stigma towards people with severe mental illness: Systematic review and meta-analysis. *Journal of Psychiatric Research,**103*, 120–133. 10.1016/j.jpsychires.2018.05.017 29843003 10.1016/j.jpsychires.2018.05.017

[CR58] Nathan, P. E., Conrad, M., & Skinstad, A. H. (2016). History of the concept of addiction. *Annual Review of Clinical Psychology,**12*(1), 29–51. 10.1146/annurev-clinpsy-021815-093546 26565120 10.1146/annurev-clinpsy-021815-093546

[CR59] Orsmond, G. I., & Cohn, E. S. (2015). The distinctive features of a feasibility study: Objectives and guiding questions. *OTJR: Occupational Therapy Journal of Research,**35*(3), 169–177. 10.1177/1539449215578649 26594739 10.1177/1539449215578649

[CR60] Penn, D. L., Guynan, K., Daily, T., Spaulding, W. D., Garbin, C. P., & Sullivan, M. (1994). Dispelling the stigma of schizophrenia: What sort of information is best? *Schizophrenia Bulletin,**20*(3), 567–578. 10.1093/schbul/20.3.567 7973472 10.1093/schbul/20.3.567

[CR61] Pescosolido, B. A., Martin, J. K., Long, J. S., Medina, T. R., Phelan, J. C., & Link, B. G. (2010). “A disease like any other”? A decade of change in public reactions to schizophrenia, depression, and alcohol dependence. *American Journal of Psychiatry,**167*(11), 1321–1330. 10.1176/appi.ajp.2010.09121743 20843872 10.1176/appi.ajp.2010.09121743 PMC4429867

[CR62] Pettigrew, T. F., & Tropp, L. R. (2006). A meta-analytic test of intergroup contact theory. *Journal of Personality and Social Psychology,**90*(5), 751–783. 10.1037/0022-3514.90.5.751 16737372 10.1037/0022-3514.90.5.751

[CR63] Pettigrew, T. F., & Tropp, L. R. (2008). How does intergroup contact reduce prejudice? Meta-analytic tests of three mediators. *European Journal of Social Psychology,**38*(6), 922–934. 10.1002/ejsp.504

[CR64] Phelan, J. C., Link, B. G., & Dovidio, J. F. (2008). Stigma and prejudice: One animal or two? *Social Science and Medicine,**67*(3), 358–367. 10.1016/j.socscimed.2008.03.022 18524444 10.1016/j.socscimed.2008.03.022 PMC4007574

[CR65] Pinto, M. D., Hickman, R., Logsdon, M. C., & Burant, C. (2012). Psychometric evaluation of the revised attribution questionnaire (r-AQ) to measure mental illness stigma in adolescents. *Journal of Nursing Measurement,**20*(1), 47–58. 10.1891/1061-3749.20.1.47 22679709 10.1891/1061-3749.20.1.47 PMC3506425

[CR66] Probst, C., Manthey, J., Martinez, A., & Rehm, J. (2015). Alcohol use disorder severity and reported reasons not to seek treatment: A cross-sectional study in European primary care practices. *Substance Abuse Treatment, Prevention, and Policy,**10*(1), 32. 10.1186/s13011-015-0028-z 26264215 10.1186/s13011-015-0028-z PMC4534056

[CR67] Pryor, J. B., & Reeder, G. D. (2011). HIV-related stigma. In C. J. Cockerell, B. J. Hall, & J. C. Hall (Eds.), *HIV/AIDS in the post-HAART era: Manifestations, treatment, and epidemiology* (pp. 790–806). People’s Medical Publishing House USA Ltd.

[CR68] Reinke, R. R., Corrigan, P. W., Leonhard, C., Lundin, R. K., & Kubiak, M. A. (2004). Examining two aspects of contact on the stigma of mental illness. *Journal of Social and Clinical Psychology,**23*(3), 377–389. 10.1521/jscp.23.3.377.35457

[CR69] Rothbart, M., & John, O. P. (1985). Social categorization and behavioral episodes: A cognitive analysis of the effects of intergroup contact. *Journal of Social Issues,**41*(3), 81–104. 10.1111/j.1540-4560.1985.tb01130.x

[CR70] Saunders, J. B., Degenhardt, L., Reed, G. M., & Poznyak, V. (2019). Alcohol use disorders in ICD-11: Past, present, and future. *Alcoholism, Clinical and Experimental Research,**43*(8), 1617–1631. 10.1111/acer.14128 31194891 10.1111/acer.14128

[CR71] Schomerus, G., Corrigan, P. W., Klauer, T., Kuwert, P., Freyberger, H. J., & Lucht, M. (2011). Self-stigma in alcohol dependence: Consequences for drinking-refusal self-efficacy. *Drug and Alcohol Dependence,**114*(1), 12–17. 10.1016/j.drugalcdep.2010.08.013 20933344 10.1016/j.drugalcdep.2010.08.013

[CR72] Schomerus, G., Matschinger, H., Lucht, M. J., & Angermeyer, M. C. (2014). Changes in the perception of alcohol-related stigma in Germany over the last two decades. *Drug and Alcohol Dependence,**143*, 225–231. 10.1016/j.drugalcdep.2014.07.033 25156226 10.1016/j.drugalcdep.2014.07.033

[CR73] Shahbaznezhad, H., Dolan, R., & Rashidirad, M. (2021). The role of social media content format and platform in users’ engagement behavior. *Journal of Interactive Marketing,**53*(1), 47–65. 10.1016/j.intmar.2020.05.001

[CR74] Skivington, K., Matthews, L., Simpson, S. A., Craig, P., Baird, J., Blazeby, J. M., Boyd, K. A., Craig, N., French, D. P., McIntosh, E., Petticrew, M., Rycroft-Malone, J., White, M., & Moore, L. (2021). A new framework for developing and evaluating complex interventions: update of Medical Research Council guidance. *BMJ,**374*, n2061. 10.1136/bmj.n2061 34593508 10.1136/bmj.n2061 PMC8482308

[CR75] Smith, S. M., Dawson, D. A., Goldstein, R. B., & Grant, B. F. (2010). Examining perceived alcoholism stigma effect on racial-ethnic disparities in treatment and quality of life among alcoholics. *Journal of Studies on Alcohol and Drugs,**71*(2), 231–236. 10.15288/jsad.2010.71.231 20230720 10.15288/jsad.2010.71.231 PMC2841733

[CR76] Thornicroft, G., Mehta, N., Clement, S., Evans-Lacko, S., Doherty, M., Rose, D., Koschorke, M., Shidhaye, R., O’Reilly, C., & Henderson, C. (2016). Evidence for effective interventions to reduce mental-health-related stigma and discrimination. *The Lancet,**387*(10023), 1123–1132. 10.1016/S0140-6736(15)00298-6 10.1016/S0140-6736(15)00298-6 26410341

[CR77] Tostes, J. G. D. A., Dias, R. T., Reis, A. A. D. S., Silveira, P. S. D., & Ronzani, T. M. (2020). Interventions to reduce stigma related to people who use drugs: Systematic review. *Paidéia (Ribeirão Preto),**30*, e3022. 10.1590/1982-4327e3022

[CR78] Vanyukov, M. (2024). The misnomer of substance use “stigma”: Beneficial disapproval should not be conflated with mistreatment of users. *Addict Res Theory,**32*(2), 101–103. 10.1080/16066359.2023.2283574 38523740 10.1080/16066359.2023.2283574 PMC10957147

[CR79] Wallhed Finn, S., Bakshi, A.-S., & Andréasson, S. (2014). Alcohol consumption, dependence, and treatment barriers: Perceptions among nontreatment seekers with alcohol dependence. *Substance Use & Misuse,**49*(6), 762–769. 10.3109/10826084.2014.891616 24601784 10.3109/10826084.2014.891616

[CR80] Wei, Y., McGrath, P. J., Hayden, J., & Kutcher, S. (2015). Mental health literacy measures evaluating knowledge, attitudes and help-seeking: A scoping review. *BMC Psychiatry,**15*(1), 291. 10.1186/s12888-015-0681-9 26576680 10.1186/s12888-015-0681-9 PMC4650294

[CR81] Weiner, B. (1986). Attribution, emotion, and action. In R. M. Sorrentino & E. T. Higgins (Eds.), *Handbook of motivation and cognition: Foundations of social behavior* (pp. 281–312). Guilford Press.

