# Supplementary material for: Challenging the Public Stigma of Alcohol Use Disorder in the UK Using Video ‘Education’ and ‘Contact’ Interventions: A Pilot Study
Source: Int J Ment Health Addict. 2024 Oct 9;24(1):66–91. doi: 10.1007/s11469-024-01393-y (PMC12886277; doi:10.1007/s11469-024-01393-y)
Supplement: Supplementary file 1 — Supplementary file1 (PDF 1441 KB) [file 11469_2024_1393_MOESM1_ESM.pdf]

Challenging the public stigma of alcohol use disorder in the UK using video 'education' and 'contact' interventions: a pilot study

Authors:

Sophie Hytner[1#], Daphne Josselin[1], David Belin[2*] & Owen Bowden Jones[2*]

Supplementary Online Materials

SOM Table I

Feasibility and Acceptability Questionnaire

This study is a pilot to prepare for a larger study into public attitudes towards alcohol dependence. To help design this study as effectively as possible, please tell us how much you agree with the following statements:

1. Briefing and questionnaires (*Objective 2: Evaluation and Refinement of Data Collection Procedures and Outcome measures)

- The initial information sheet gave me a clear explanation of what taking part in the study would involve

Strongly Disagree	Disagree	Neither Agree Nor Disagree	Agree	Strongly Agree
☐	☐	☐	☐	☐

- The initial instructions and briefing on the questionnaires meant I understood what was being asked of me

Strongly Disagree	Disagree	Neither Agree Nor Disagree	Agree	Strongly Agree
☐	☐	☐	☐	☐

- The questions and statements on the questionnaires were clear and easy to understand

Strongly Disagree	Disagree	Neither Agree Nor Disagree	Agree	Strongly Agree
☐	☐	☐	☐	☐

- I was able to accurately record my feelings towards John using the scoring scale provided on the questionnaires

Strongly Disagree	Disagree	Neither Agree Nor Disagree	Agree	Strongly Agree
☐	☐	☐	☐	☐

- The time it took me to fill out the questionnaires was:

Too short	About right	Too long
☐	☐	☐

2. Video content and format (*Objective 3: Evaluation of Acceptability and Suitability of Intervention and Study Procedures)

- The video was clear and easy to follow

Strongly Disagree	Disagree	Neither Agree Nor Disagree	Agree	Strongly Agree
☐	☐	☐	☐	☐

- The content in the video was engaging and interesting

Strongly Disagree	Disagree	Neither Agree Nor Disagree	Agree	Strongly Agree
☐	☐	☐	☐	☐

- The content in the video felt relevant to me

Strongly Disagree	Disagree	Neither Agree Nor Disagree	Agree	Strongly Agree
☐	☐	☐	☐	☐

- I learned information that I didn't previously know

Strongly Disagree	Disagree	Neither Agree Nor Disagree	Agree	Strongly Agree
☐	☐	☐	☐	☐

- I would choose to watch this video in full in my own time

Strongly Disagree	Disagree	Neither Agree Nor Disagree	Agree	Strongly Agree
☐	☐	☐	☐	☐

- The video length was:

Too short	About right	Too long
☐	☐	☐

(*Objective 5: Preliminary Evaluation of Participant Responses to Intervention)

What did you like about the video?

[free text]

How could the video be improved?

[free text]

SOM Table II

A) Myths and facts used in the educational video

Category	Myths	Facts
**Cannot recover**	People can't recover from alcohol dependence	People can recover from alcohol dependence and go on to lead fulfilling lives.<br>⍰<br>Over half of people in treatment for alcohol use in alcohol services in England stop or reduce their drinking after 6 months of treatment.
**Only affects certain groups**	Alcohol dependence only affects certain types of people, like the homeless	Anyone can become dependent on alcohol, regardless of their age, gender, ethnicity or background.<br><br>Most people with alcohol dependence in England are in employment and stable housing.
**To blame**	People with alcohol dependence are to blame for their problems	A person's risk of developing alcohol dependence is influenced by lots of biological, psychological and social factors, many of which are outside their control.<br><br>Biological factors can include a person's genetics – approximately 50% of the risk for developing alcohol dependence is explained by genes.<br><br>Psychological factors can include mental health problems (like depression or anxiety) and personality traits (like impulsivity).<br><br>Social factors can include trauma (like childhood abuse); social isolation; poverty; unemployment; or discrimination.<br><br>These factors influence each other and can combine to increase someone's chance of becoming alcohol dependent.
**Able to control**	People with alcohol dependence could stop or control their drinking if they wanted to	Alcohol dependence leads to changes in the brain that can limit a person's control over their drinking.<br><br>For example, when a person becomes alcohol dependent, their brain adapts to heavy alcohol use and can start to need alcohol to maintain its chemical balance.<br><br>If a person with alcohol dependence stops drinking, this balance can be disrupted and the person may experience harmful symptoms, like anxiety, shaking or seizures.<br><br>This means that while many people with alcohol dependence try very hard (often repeatedly) to stop drinking, it can be very difficult, and in some cases unsafe, for them to do so without support.
**Bad character**	People with alcohol dependence don't care about others	Those with alcohol dependence tend to feel guilty and ashamed about the impact of their actions on other people; and caring about others is a key factor that can lead people to seek treatment.<br><br>A large number of people with alcohol dependence support other people – 81% of those attending Alcoholics Anonymous groups in the UK volunteer their time to help others.

B) Slides from the educational video

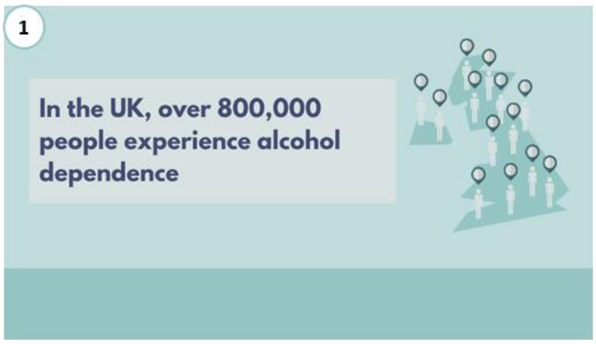

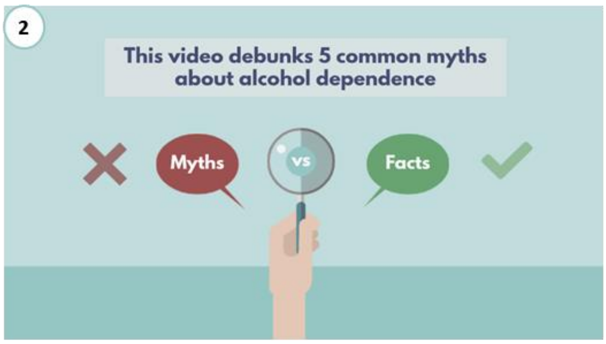

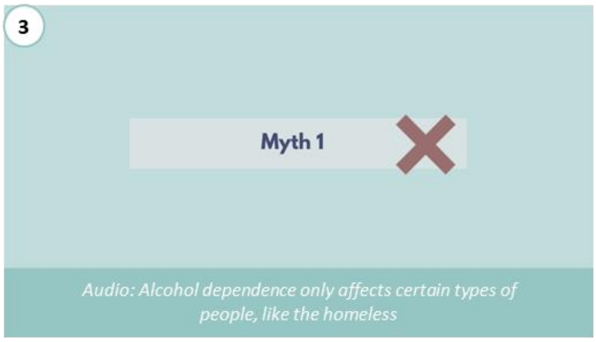

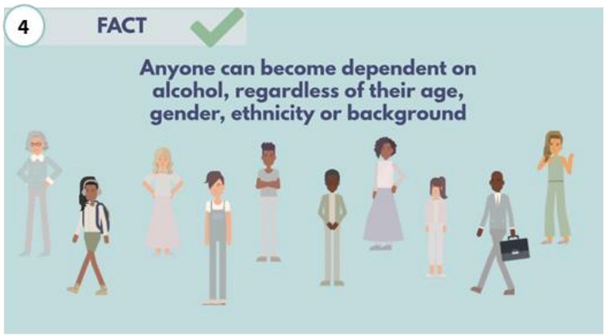

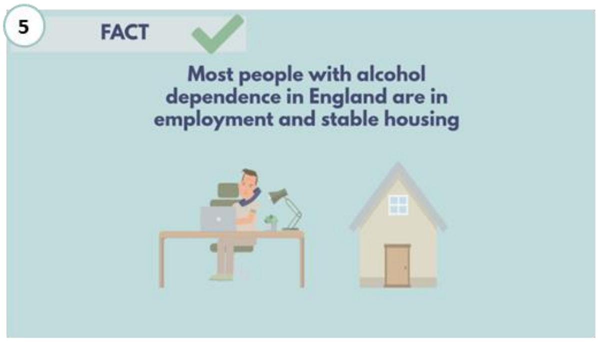

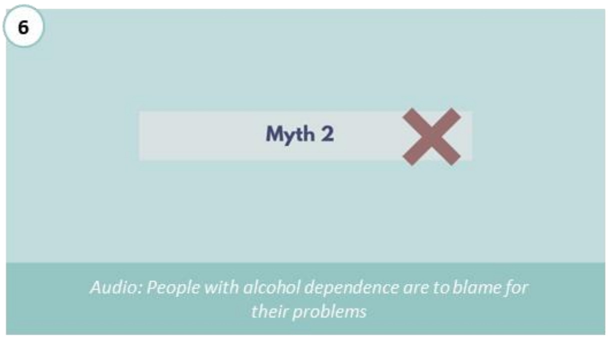

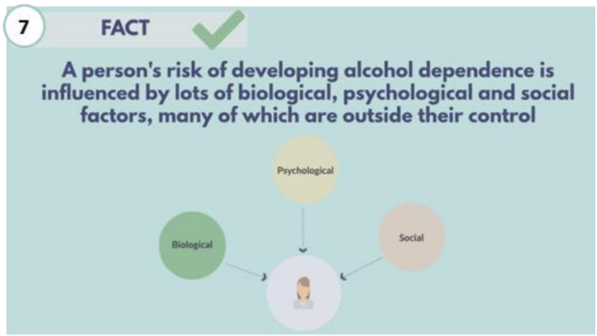

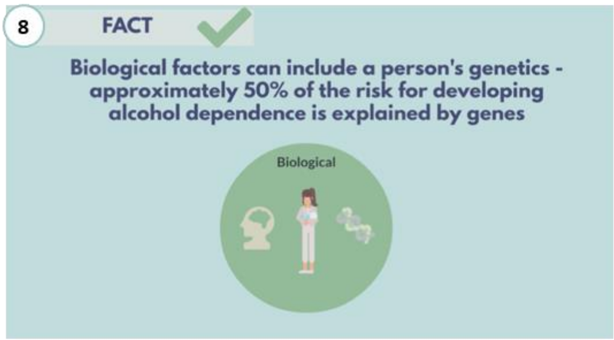

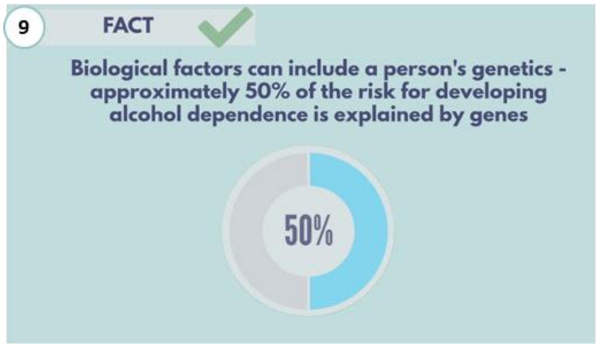

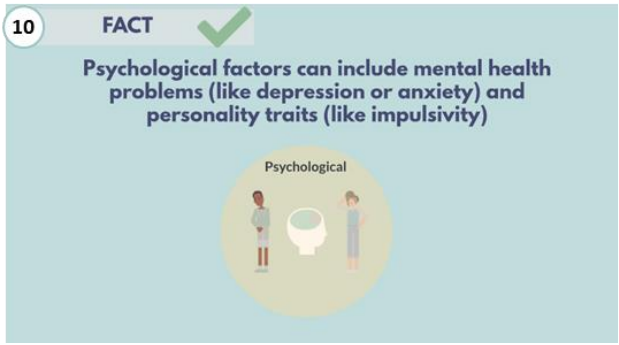

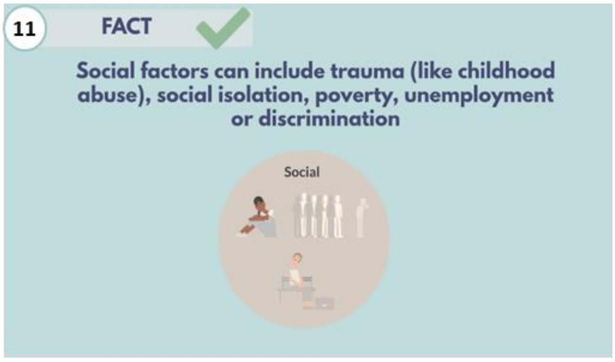
11
FACT
Social factors can include trauma (like childhood abuse), social isolation, poverty, unemployment or discrimination
Social

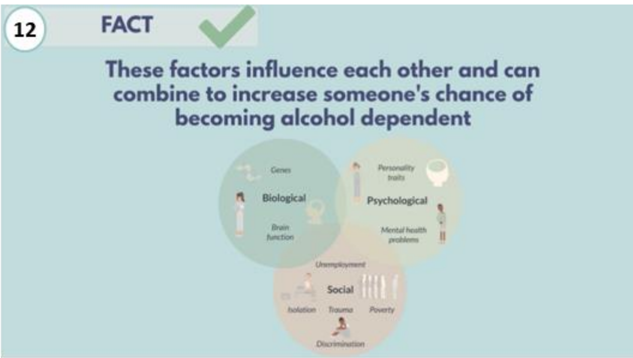
12
FACT
These factors influence each other and can combine to increase someone's chance of becoming alcohol dependent
Genes
Biological
Brain function
Personality traits
Psychological
Mental health problems
Unemployment
Social
Isolation
Trauma
Poverty
Discrimination

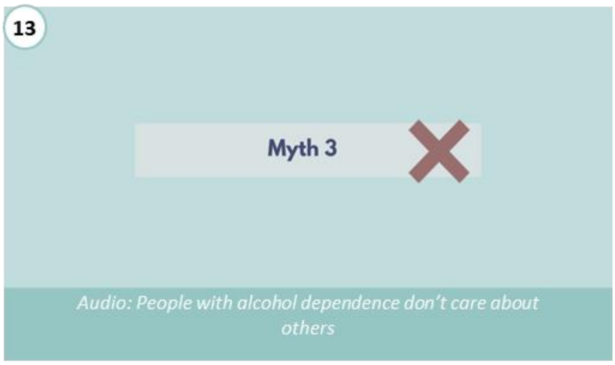
13
Myth 3
Audio: People with alcohol dependence don't care about others

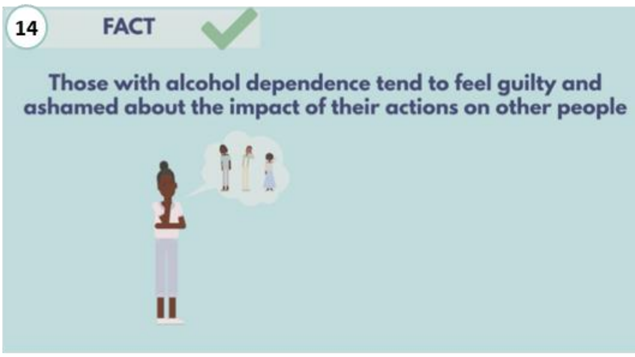
14
FACT
Those with alcohol dependence tend to feel guilty and ashamed about the impact of their actions on other people

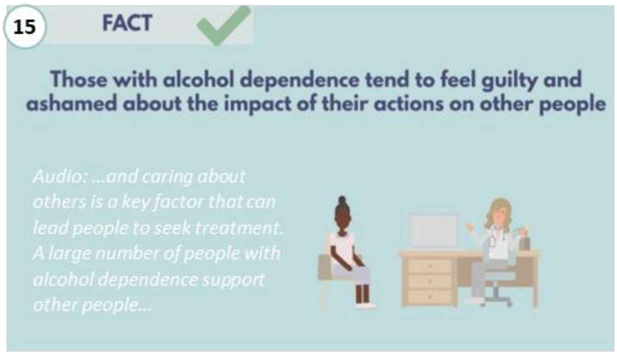
15
FACT
Those with alcohol dependence tend to feel guilty and ashamed about the impact of their actions on other people
Audio: ...and caring about others is a key factor that can lead people to seek treatment. A large number of people with alcohol dependence support other people...

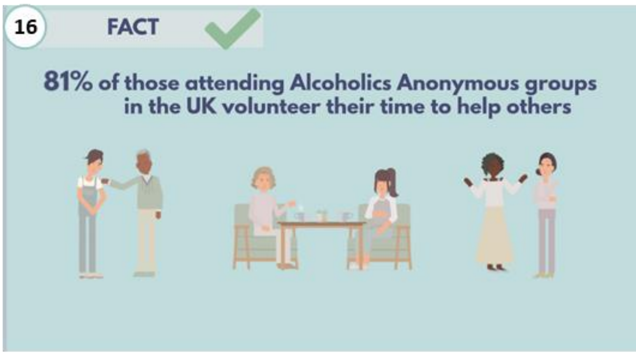
16
FACT
81% of those attending Alcoholics Anonymous groups in the UK volunteer their time to help others

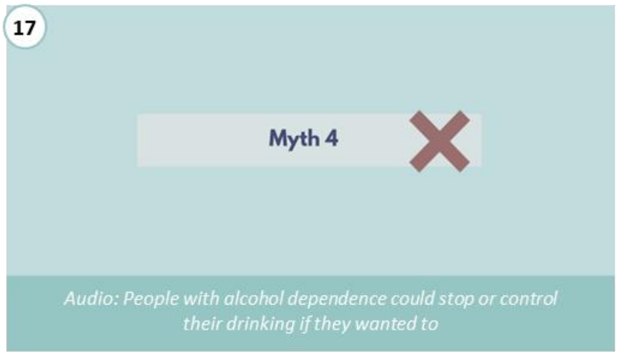
17
Myth 4
Audio: People with alcohol dependence could stop or control their drinking if they wanted to

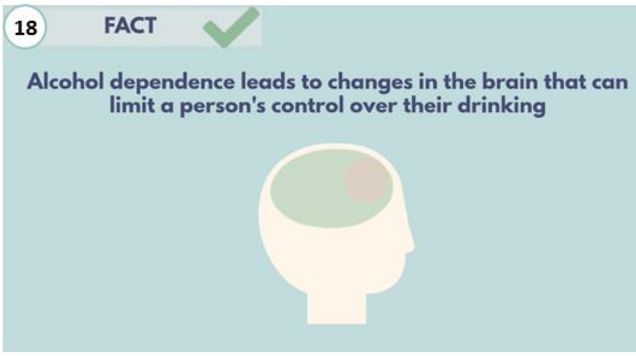
18
FACT
Alcohol dependence leads to changes in the brain that can limit a person's control over their drinking

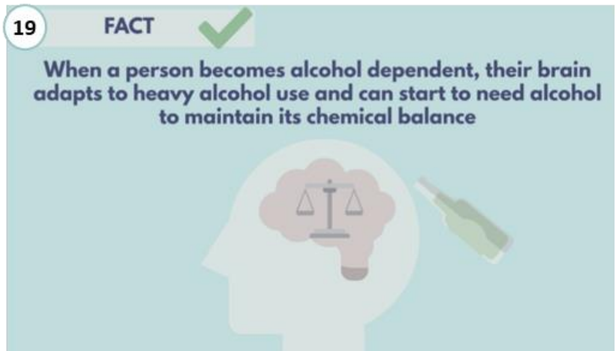
19
FACT
When a person becomes alcohol dependent, their brain adapts to heavy alcohol use and can start to need alcohol to maintain its chemical balance

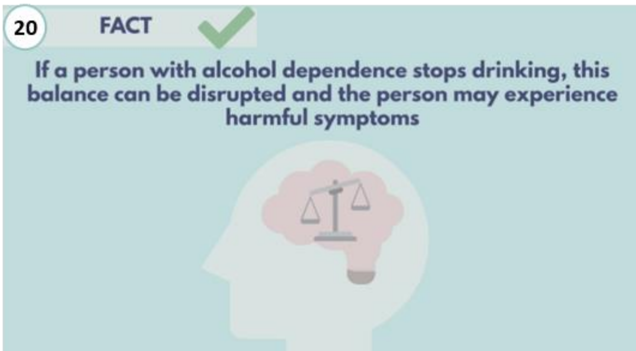
20
FACT
If a person with alcohol dependence stops drinking, this balance can be disrupted and the person may experience harmful symptoms

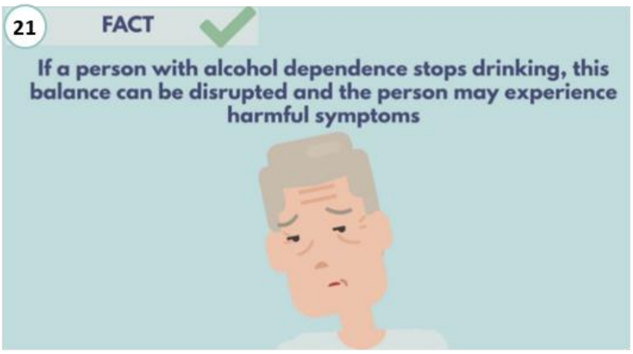
21
FACT
If a person with alcohol dependence stops drinking, this balance can be disrupted and the person may experience harmful symptoms

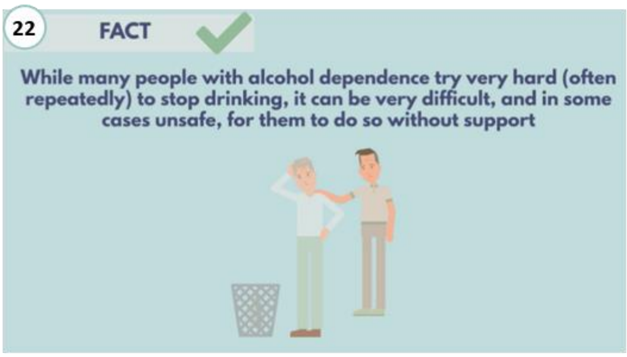
22
FACT
While many people with alcohol dependence try very hard (often repeatedly) to stop drinking, it can be very difficult, and in some cases unsafe, for them to do so without support

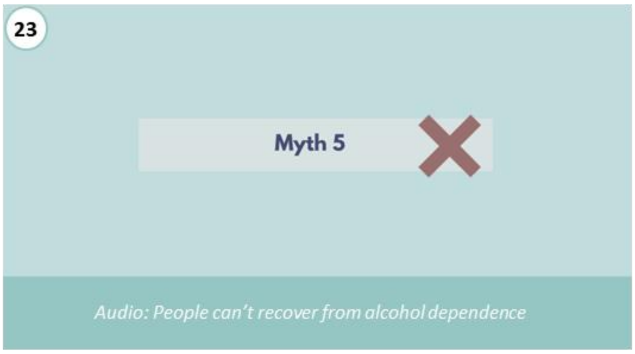
23
Myth 5
Audio: People can't recover from alcohol dependence

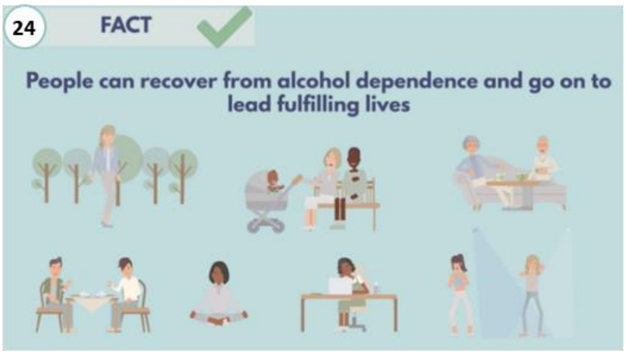
24
FACT
People can recover from alcohol dependence and go on to lead fulfilling lives

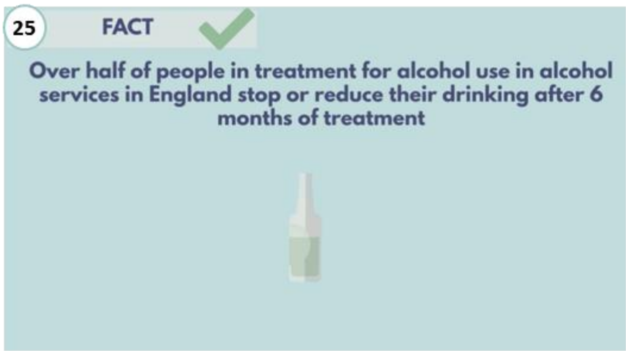
25
FACT
Over half of people in treatment for alcohol use in alcohol services in England stop or reduce their drinking after 6 months of treatment

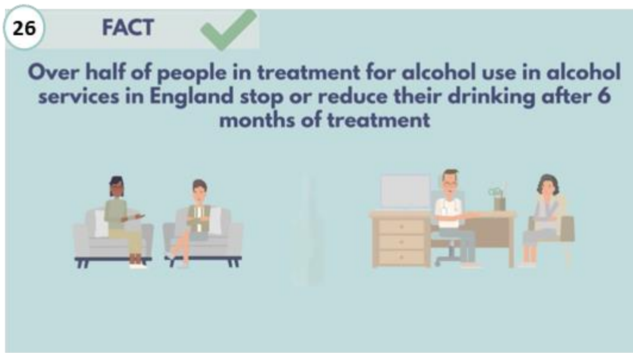
26
FACT
Over half of people in treatment for alcohol use in alcohol services in England stop or reduce their drinking after 6 months of treatment

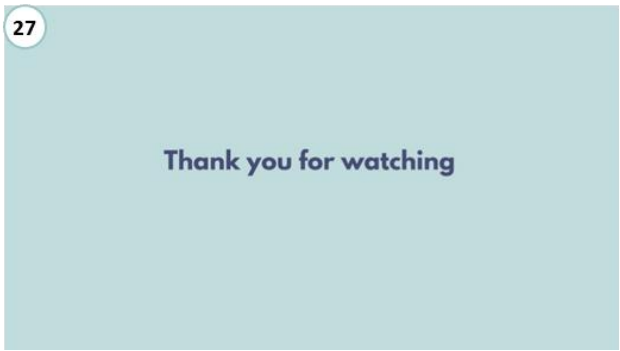
27
Thank you for watching

SOM Table III

Interview guide and content for contact video

Themes	Prompts	Interview Questions	Included interview content
**Section 1**			
**Introductions**			**Slide 1 (black background with white writing): In the UK, approximately 800,000 people experience alcohol dependence**<br><br>**Slide 2: In this video, three people speak about their personal experiences of alcohol dependence**
	Name	How would you like to introduce yourself?	My name is X; I'm Y; I'm Z
	Diagnosis	How would you describe your past relationship with alcohol? E.g. alcoholic, dependent on alcohol	X: I was dependent on alcohol and I'd like to say I'm not dependent on alcohol anymore<br><br>Y: I'm recovering from alcoholism...I've been completely abstinent now for four and a half years<br><br>Z: I would class myself as someone who was dependent on alcohol and nowadays I don't drink it at all, I don't think about it very much and my life is very much devoid of alcohol dependence
	Recovery status	How would you describe your current relationship with alcohol? E.g. sober, in recovery for x years	
**Background**			**Heading: How it started**
	Aetiology (e.g. early experiences, first experiences of using substance)	What do you believe led you to use alcohol?	Y: My alcohol use was basically an escape from my reality because of trauma that I experienced in childhood<br><br>X: My mum left home when I was 14 years old, my dad met someone else and I felt incredibly lonely and rejected<br><br>Z: I was quite shy and introverted - drinking alcohol gave me confidence, it made me feel as though I had a greater ability to be more open with my emotions
	Function	In your view, what did using alcohol give you / what problems did it seem to solve?<br>What do you think kept your alcohol use going?	

Themes	Prompts	Interview Questions	Included interview content
**Symptoms**			**Heading: What it was like**
	First experience and progression of symptoms (e.g. increased use, withdrawal symptoms, preoccupation)	What difficulties did you experience as a result of your alcohol use?	Y: When I first started drinking, it was a recreational thing. There were lots of difficulties that came as my drinking progressed<br><br>X: I lost all my self esteem, my anxiety was sky high<br><br>Z: I was almost always sick, you know, low moods, feelings of like self loathing<br><br>X: It got to quite a critical state really where I was drinking over a litre of vodka a night at times<br><br>Y: I ended up becoming homeless
**Challenges**	*Impact of condition (e.g. distress, stigma, impairments in occupational and interpersonal functioning)*	What challenges / problems did this lead to?	
**Section 2**			
**Acceptance / Treatment**			**Heading: Getting help**
	Recognising problem, contemplating and seeking treatment	What led you to seek help with your alcohol use?	Z: What kind of woke me up was I had a health scare - it really shook me up. That same year I actually went for some talking therapy with my local authority. Those two things really like planted a seed<br><br>X: I found my community actually on Instagram and I went to a couple of sober social things that I was petrified to go to, and it was there that I met some really amazing people actually - there's so much support out there<br><br>Y: Finally, what got me and kept me sober was a combination of a twelve step fellowship and um some really intense trauma therapy
	Engaging in treatment (specific approaches)	What support did you receive? (E.g. treatment, self-help groups). How long did this last?	
	Learning of coping strategies	How was this helpful for you / what did this give you / what did you learn?	

Themes	Prompts	Interview Questions	Included interview content
**Recovery**			**<u>Heading: What I've gained</u>**
	Greater control over symptoms	What happened to your drinking after that / what changed?	Z: What I've gained from not drinking is all of the things that I got from drinking but from within myself. I don't go out to get smashed - I go out to spend time with people<br><br>Y: I quite like mornings - I get up and you know I now run, I love being outdoors<br><br>X: I've got a lot more energy, I mean I have a personal trainer 3 times a week now - I would have not even thought about that before, you know, I've lost two stone. My relationships with the family and my wife has improved<br><br>Z: I would definitely give myself a pat on the back for being an amazing father. I meditate quite a lot and that's also allowed me to sort of build resilience around the rigors of just everyday life
	Recovery journey	How would you describe your recovery journey?	
		What benefits did you experience in the process of recovery?	
		What challenges occurred in the recovery process?	
		How did you overcome these / what helped?	
	Meaningful and satisfactory quality of life	How is your life different now compared to when you were drinking? What has changed?	
	Examples (e.g. living independently, improved relationships, increased community activities and hobbies)	What do you enjoy about life without drinking?	
**Achievements**			**<u>Heading: What life is like now</u>**
	Successes in recovery	What have you achieved that you're proud of as a result of not drinking? E.g. work, hobbies, community activities, personal progress, relationships with others	Y: I'm now a qualified trauma and addiction recovery coach. I think what recovery has done is it's given me the ability to do the job that I do now which is to, to help other people<br><br>Z: I'm a music journalist - I've done a lot of reviews of festivals, club events, gigs<br><br>X: I've created my own podcast called 'W' and that's been in the top 10 of the Apple charts. I'm now a qualified coach so I help people stop drinking as well
		What other successes / strengths have you gained through your recovery?	

Themes	Prompts	Interview Questions	Included interview content
**Section 3**			
**Ongoing challenges**			**<u>Heading: What I have learned</u>**
	Challenges and symptoms during recovery	Is there anything you continue to struggle with? If so, what helps you with this?	X: It's not all bells and whistles - there are times that I think it would be nice to have a drink<br><br>Z: If you're tired or you're feeling a bit cranky then it's difficult to pull yourself out of that mode, but you learn to accept your emotions rather than suppressing them<br><br>Y: One of the biggest things I've learned through my recovery is that I can live through pain - what's waiting on the other side of that pain is really something amazing
**Hope**	*Possible to live with the condition despite challenges (uplifting tone)*	How would you describe your overall quality of life now compared to when you were drinking?	Y: It's like another world - it's another life
**Closing**	*Contributors insights*	Is there something you would particularly like people to know about alcohol dependence or your experiences that wasn't covered during this interview?	Y: What I would want to say to people is look underneath - don't ask why the addiction, ask why the pain<br><br>X: If you're a relative or a family member or a loved one, there is support out there, and you need to talk about it and share your experience and there is support out there so reach out for that
			**Final slide: Thank you for watching**

SOM Figure 1

Vignette used with Attribution Questionnaire-27 (AQ-27) and Social Distance Scale (SDS)

During the last month, John has started to drink more than his usual amount of alcohol. In fact, he has noticed that he needs to drink twice as much as he used to in order to get the same effect. Several times, he has tried to cut down, or stop drinking, but he can't. Each time he has tried to cut down, he became very agitated, sweaty, and he couldn't sleep, so he took another drink. His family has complained that he is often hungover, and has become unreliable—making plans one day, and cancelling them the next.

SOM Figure 2

Outliers as assessed by Box Plots

SDS

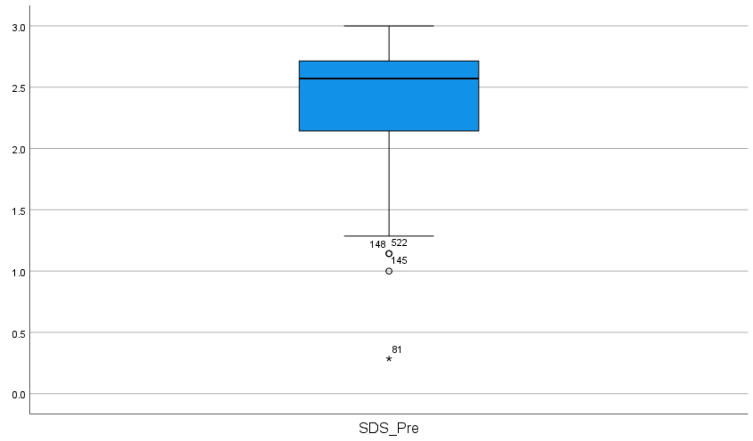

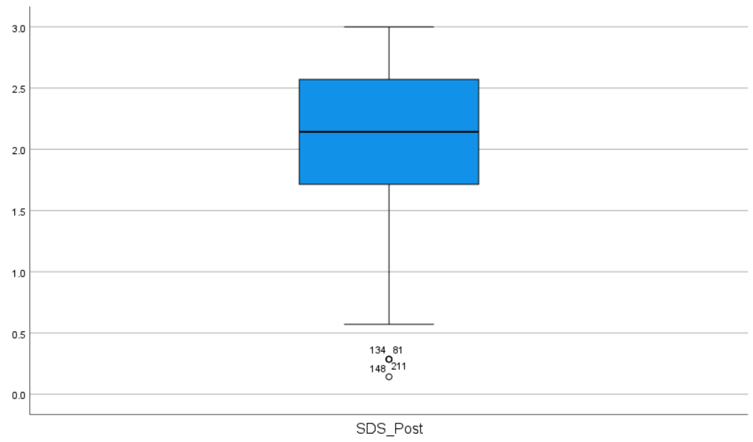

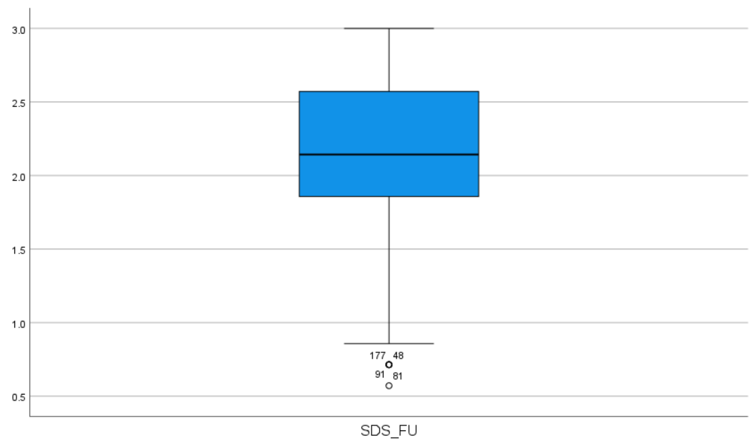

AQ

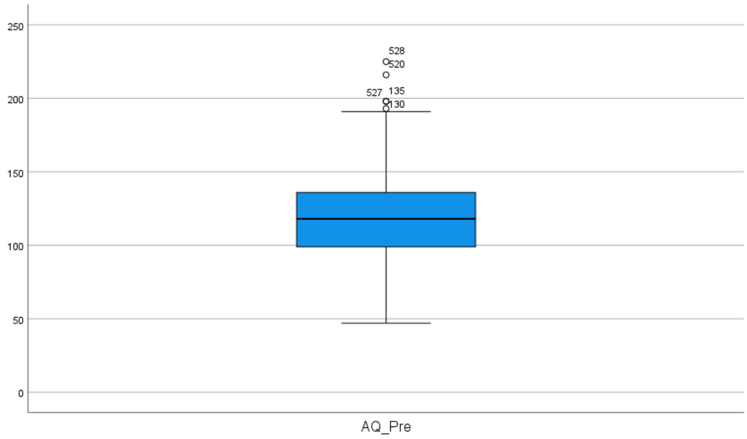

250
200
150
100
50
0
528
520
527
135
130
AQ_Pre

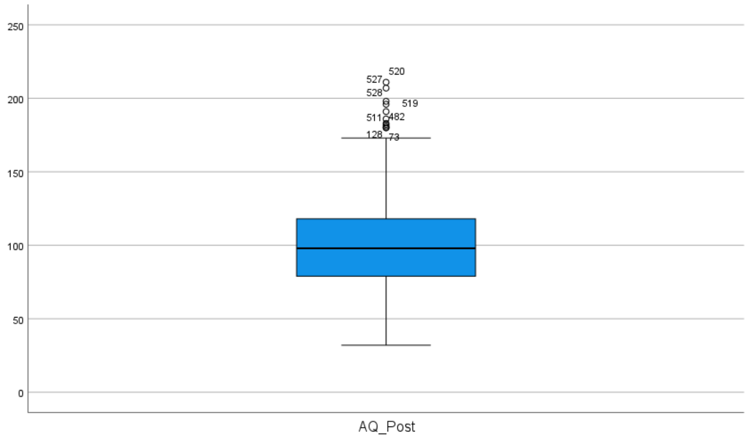

250
200
150
100
50
0
520
527
528
519
511
482
128
73
AQ_Post

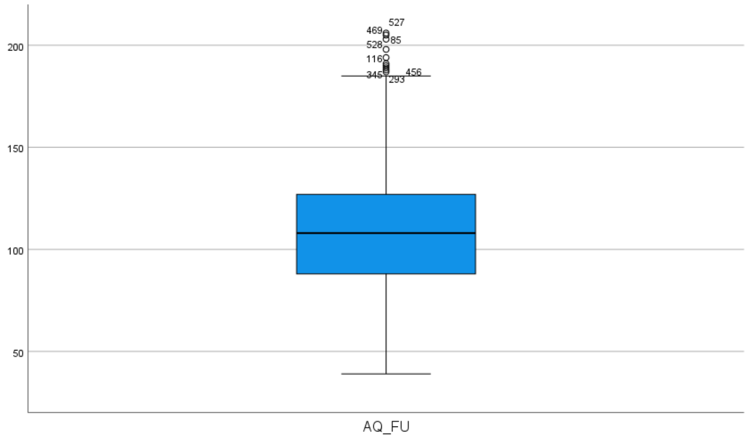

200
150
100
50
527
469
85
528
116
456
345
293
AQ_FU

SOM Figure 3

Tests of Normality

Shapiro Wilk

Tests of Normality

	Kolmogorov-Smirnov[a]			Shapiro-Wilk		
	Statistic	df	Sig.	Statistic	df	Sig.
SDS_Pre	.138	539	<.001	.935	539	<.001
SDS_Post	.106	539	<.001	.964	539	<.001
SDS_FU	.095	539	<.001	.967	539	<.001
AQ_Pre	.036	539	.085	.992	539	.005
AQ_Post	.058	539	<.001	.976	539	<.001
AQ_FU	.043	539	.021	.987	539	<.001

a. Lilliefors Significance Correction

Histograms

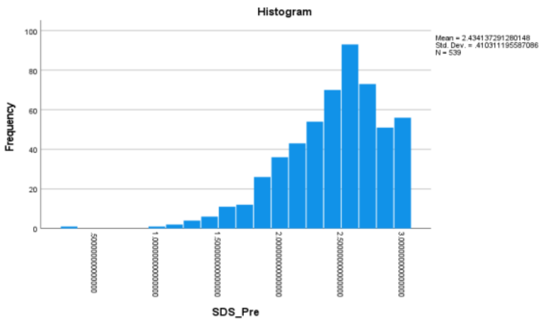

Histogram
Mean = 2.43413729128014 8
Std. Dev. = .410311195587086
N = 539
Frequency
0
20
40
60
80
100
SDS_Pre

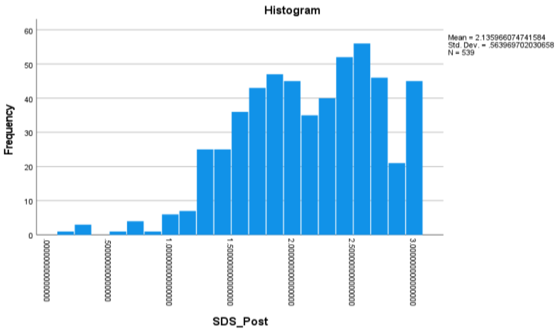

Histogram
Mean = 2.135966074741584
Std. Dev. = .563969702030658
N = 539
Frequency
0
10
20
30
40
50
60
SDS_Post

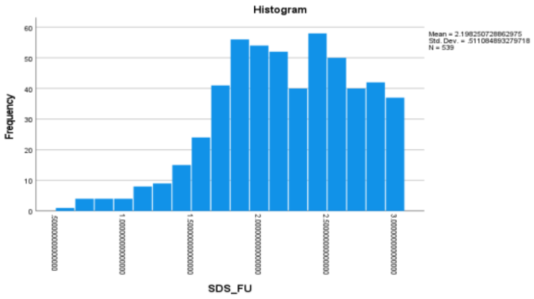

Histogram
Mean = 2.198250728862975
Std. Dev. = .511084893279718
N = 539
Frequency
0
10
20
30
40
50
60
SDS_FU

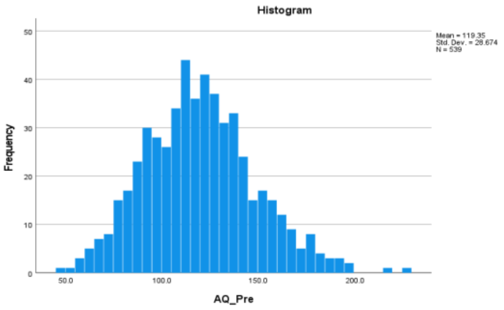

Histogram
Mean = 119.35
Std. Dev. = 28.674
N = 539
Frequency
0
10
20
30
40
50
50.0
100.0
150.0
200.0
AQ_Pre

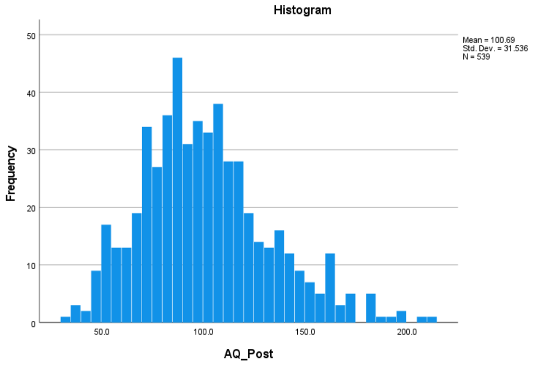

Histogram
Mean = 100.69
Std. Dev. = 31.536
N = 539
Frequency
0
10
20
30
40
50
50.0
100.0
150.0
200.0
AQ_Post

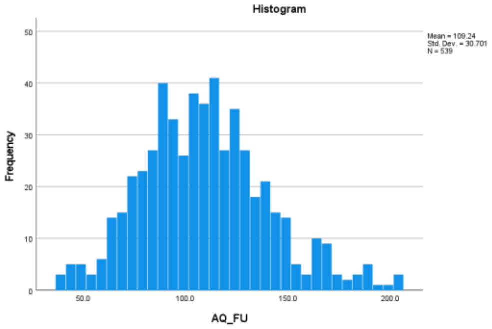

Histogram
Mean = 109.24
Std. Dev. = 30.701
N = 539
Frequency
0
10
20
30
40
50
50.0
100.0
150.0
200.0
AQ_FU

Skewness

Descriptives

			Statistic	Std. Error
SDS_Pre	Mean		2.434137291280148	.017673354325253
	95% Confidence Interval for Mean	Lower Bound	2.399420051335952	
		Upper Bound	2.468854531224345	
	5% Trimmed Mean		2.459772653650205	
	Median		2.571428571428572	
	Variance		.168	
	Std. Deviation		.410311195587086	
	Minimum		.285714285714286	
	Maximum		3.000000000000000	
	Range		2.714285714285715	
	Interquartile Range		.571428571428572	
	Skewness		-.924	.105
	Kurtosis		1.350	.210
SDS_Post	Mean		2.135966074741585	.024291894737198
	95% Confidence Interval for Mean	Lower Bound	2.088247485345942	
		Upper Bound	2.183684664137228	
	5% Trimmed Mean		2.156403687015931	
	Median		2.142857142857143	
	Variance		.318	
	Std. Deviation		.563969702030658	
	Minimum		.142857142857143	
	Maximum		3.000000000000000	
	Range		2.857142857142857	
	Interquartile Range		.857142857142857	
	Skewness		-.449	.105
	Kurtosis		-.124	.210
SDS_FU	Mean		2.198250728862974	.022013984766593
	95% Confidence Interval for Mean	Lower Bound	2.155006827495725	
		Upper Bound	2.241494630230223	
	5% Trimmed Mean		2.218835586182525	

	Median		2.142857142857143	
	Variance		.261	
	Std. Deviation		.511084893279718	
	Minimum		.571428571428571	
	Maximum		3.000000000000000	
	Range		2.428571428571429	
	Interquartile Range		.714285714285714	
	Skewness		-.423	.105
	Kurtosis		-.180	.210
AQ_Pre	Mean		119.353	1.2351
	95% Confidence Interval for Mean	Lower Bound	116.926	
		Upper Bound	121.779	
	5% Trimmed Mean		118.694	
	Median		118.000	
	Variance		822.225	
	Std. Deviation		28.6745	
	Minimum		47.0	
	Maximum		225.0	
	Range		178.0	
	Interquartile Range		37.0	
	Skewness		.354	.105
	Kurtosis		.180	.210
AQ_Post	Mean		100.694	1.3584
	95% Confidence Interval for Mean	Lower Bound	98.026	
		Upper Bound	103.362	
	5% Trimmed Mean		99.464	
	Median		98.000	
	Variance		994.547	
	Std. Deviation		31.5364	
	Minimum		32.0	
	Maximum		211.0	
	Range		179.0	
	Interquartile Range		39.0	
	Skewness		.610	.105
	Kurtosis		.354	.210
AQ_FU	Mean		109.237	1.3224

	95% Confidence Interval for Mean	Lower Bound	106.640	
		Upper Bound	111.835	
	5% Trimmed Mean		108.383	
	Median		108.000	
	Variance		942.549	
	Std. Deviation		30.7010	
	Minimum		39.0	
	Maximum		206.0	
	Range		167.0	
	Interquartile Range		39.0	
	Skewness		.417	.105
	Kurtosis		.248	.210

Transformed Shapiro-Wilk

SDS

Tests of Normality

	Kolmogorov-Smirnov[a]			Shapiro-Wilk		
	Statistic	df	Sig.	Statistic	df	Sig.
SDSPreModerateT	.120	539	<.001	.959	539	<.001
SDSPostModerateT	.088	539	<.001	.973	539	<.001
SDSFUModerateT	.082	539	<.001	.975	539	<.001

a. Lilliefors Significance Correction

AQ

Tests of Normality

	Kolmogorov-Smirnov[a]			Shapiro-Wilk		
	Statistic	df	Sig.	Statistic	df	Sig.
AQPreModerateT	.026	539	.200*	.999	539	.975
AQPostModerateT	.030	539	.200*	.996	539	.149
AQFUModerateT	.022	539	.200*	.997	539	.334

*. This is a lower bound of the true significance.

a. Lilliefors Significance Correction

SOM Figure 4

Levene's Tests of Equality of Error Variances

SDS

Levene's Test of Equality of Error Variances

		Levene Statistic	df1	df2	Sig.
SDS_Pre	Based on Mean	5.278	3	535	.001
	Based on Median	4.038	3	535	.007
	Based on Median and with adjusted df	4.038	3	500.298	.007
	Based on trimmed mean	4.734	3	535	.003
SDS_Post	Based on Mean	6.240	3	535	<.001
	Based on Median	6.163	3	535	<.001
	Based on Median and with adjusted df	6.163	3	502.590	<.001
	Based on trimmed mean	6.460	3	535	<.001
SDS_FU	Based on Mean	.921	3	535	.430
	Based on Median	.904	3	535	.439
	Based on Median and with adjusted df	.904	3	508.647	.439
	Based on trimmed mean	.934	3	535	.424

Tests the null hypothesis that the error variance of the dependent variable is equal across groups.[a]
a. Design: Intercept + Group
Within Subjects Design: Time

AQ

Levene's Test of Equality of Error Variances[a]

		Levene Statistic	df1	df2	Sig.
AQPreModerateT	Based on Mean	1.236	3	535	.296
	Based on Median	1.178	3	535	.317
	Based on Median and with adjusted df	1.178	3	528.848	.317
	Based on trimmed mean	1.238	3	535	.295
AQPostModerateT	Based on Mean	3.608	3	535	.013
	Based on Median	3.561	3	535	.014
	Based on Median and with adjusted df	3.561	3	525.950	.014
	Based on trimmed mean	3.599	3	535	.013
AQFUModerateT	Based on Mean	2.679	3	535	.046
	Based on Median	2.631	3	535	.049
	Based on Median and with adjusted df	2.631	3	518.960	.049
	Based on trimmed mean	2.689	3	535	.046

Tests the null hypothesis that the error variance of the dependent variable is equal across groups.
a. Design: Intercept + Group
Within Subjects Design: Time

SOM Figure 5

Mauchly's Tests of Sphericity

SDS

Mauchly's Test of Sphericity[a]

Measure: SDS

Within Subjects Effect	Mauchly's W	Approx. Chi-Square	df	Sig.	Epsilon[b] Greenhouse-Geisser
Time	.960	22.012	2	<.001	.961

Mauchly's Test of Sphericity[a]

Measure: SDS

Within Subjects Effect	Epsilon	
	Huynh-Feldt	Lower-bound
Time	.970	.500

AQ

Mauchly's Test of Sphericity[a]

Measure: AQTr

Within Subjects Effect	Mauchly's W	Approx. Chi-Square	df	Sig.	Epsilon[b]		
					Greenhouse-Geisser	Huynh-Feldt	Lower-bound
Time	.915	47.201	2	<.001	.922	.930	.500

Tests the null hypothesis that the error covariance matrix of the orthonormalized transformed dependent variables is proportional to an identity matrix.
a. Design: Intercept + Group
Within Subjects Design: Time
